# Cell cycle dynamics during diapause entry and exit in an annual killifish revealed by FUCCI technology

**DOI:** 10.1186/s13227-019-0142-5

**Published:** 2019-11-08

**Authors:** Luca Dolfi, Roberto Ripa, Adam Antebi, Dario Riccardo Valenzano, Alessandro Cellerino

**Affiliations:** 1 Max Planck Institute for the Biology of Ageing, Cologne, Germany; 2 Bio@SNS, Scuola Normale Superiore, Pisa, Italy; 3 Leibniz Institute on Aging, Fritz Lipmann Institute, Jena, Germany; 4 Present Address: Max Planck Institute for the Biology of Ageing, Cologne, Germany

## Abstract

**Background:**

Annual killifishes are adapted to surviving and reproducing over alternating dry and wet seasons. During the dry season, all adults die and desiccation-resistant embryos remain encased in dry mud for months or years in a state of diapause where their development is halted in anticipation of the months that have to elapse before their habitats are flooded again. Embryonic development of annual killifishes deviates from canonical teleost development. Epiblast cells disperse during epiboly, and a “dispersed phase” precedes gastrulation. In addition, annual fish have the ability to enter diapause and block embryonic development at the dispersed phase (diapause I), mid-somitogenesis (diapause II) and the final phase of development (diapause III). Developmental transitions associated with diapause entry and exit can be linked with cell cycle events. Here we set to image this transition in living embryos.

**Results:**

To visibly explore cell cycle dynamics during killifish development in depth, we created a stable transgenic line in *Nothobranchius furzeri* that expresses two fluorescent reporters, one for the G_1_ phase and one for the S/G_2_ phases of the cell cycle, respectively (Fluorescent Ubiquitination-based Cell Cycle Indicator, FUCCI). Using this tool, we observed that, during epiboly, epiblast cells progressively become quiescent and exit the cell cycle. All embryos transit through a phase where dispersed cells migrate, without showing any mitotic activity, possibly blocked in the G_1_ phase (diapause I). Thereafter, exit from diapause I is synchronous and cells enter directly into the S phase without transiting through G_1_. The developmental trajectories of embryos entering diapause and of those that continue to develop are different. In particular, embryos entering diapause have reduced growth along the medio-lateral axis. Finally, exit from diapause II is synchronous for all cells and is characterized by a burst of mitotic activity and growth along the medio-lateral axis such that, by the end of this phase, the morphology of the embryos is identical to that of direct-developing embryos.

**Conclusions:**

Our study reveals surprising levels of coordination of cellular dynamics during diapause and provides a reference framework for further developmental analyses of this remarkable developmental quiescent state.

## Background

Annual killifishes inhabit temporary habitats that are subject to periodic desiccations [1]. In order to survive these extreme conditions, their eggs are laid in the soft substrate and remain encased in the dry mud where they are relatively protected from desiccation and can survive for prolonged periods during the dry season and regulate their development in anticipation of the ensuing rainy season. When their habitats are flooded, these embryos hatch, grow and mature rapidly and spawn the next generation before water evaporates [2–6]. This seasonal life cycle comprising embryonic arrest is widespread in arthropods from temperate climates, but it is unique among vertebrates.

As an adaptation to seasonal water availability, embryonic development of annual killifishes deviates from canonical teleost development for three main distinctive traits. The first is a slow cell cycle during early cleavage. While embryos of non-annual teleost fishes execute one cell division every 15–30 min during the first divisions after fertilization, the rate of early cell division in annual killifishes can reach almost 2 h [7]. As a result, an annual killifish embryo can be still in the blastula stage, while a non-annual killifish embryo fertilized at the same time has started somitogenesis.

The second trait is the dispersion of epiblast cells during epiboly and a decoupling between epiboly and gastrulation. When epiboly starts, the epiblast cells delaminate, assume an amoeboid shape and migrate towards the other pole of the egg. This migration is physically guided by the spreading of the extra embryonic enveloping layer [8]. In annual killifishes, the embryo at the end of epiboly consists only of extraembryonic structures and separated epiblast cells that migrate randomly over the yolk surface in a unique developmental stage named dispersed phase [6]. The dispersed phase can last for several days, and the embryonic axis is formed by migration of the epiblast cells towards a point where they reaggregate and form the embryonic primordium. This peculiar stage is named reaggregation phase [6]. In several teleosts, including zebrafish, gastrulation and axis formation take place during epiboly. However, in annual killifishes the formation of the three embryonic layers, which happens during gastrulation, takes place after epiboly during the late aggregation phase as demonstrated by live cell imaging and by the expression of the blastopore markers *goosecoid* and *brachyury* [9].

The third unique feature of annual killifish development is the ability to enter diapause. Diapause is a state of dormancy that retards or blocks embryonic development in anticipation of predictable cyclic hostile conditions. Diapause is widespread among arthropods from temperate climates that spend in diapause the coldest part of the year. Annual killifish embryo can arrest in diapause in three specific phases of development: during the dispersed phase (diapause I), at mid-somitogenesis when most organs are formed (diapause II) or at the final stage of development (diapause III) [4]. Duration of diapause is highly variable, and diapauses are not obligatory [4]. These adaptations are interpreted as bet-hedging strategies that ensure survival in an unpredictable environment, which are typical of seed banks [3, 10]. Under appropriate conditions, such as high temperature or under the influence of maternal factors, embryos can greatly shorten and possibly skip all three diapauses and proceed through direct development [11–14]. Diapause II is not a simply a phase of developmental arrest, but direct development and diapause are alternative developmental trajectories, characterized by different morphologies. In particular, during somitogenesis, the embryos committed to diapause II grow in the longitudinal direction but are impaired in transversal growth and therefore have reduced transversal diameter of head and body as compared to direct development embryos [13, 15]. This difference is detectable already at the start of somitogenesis, and it is observed in multiple independent clades that have evolved diapause [15] and can have an impact on post-hatch life-history traits [10].

One species of annual killifish has recently become a relatively widespread experimental model: *Nothobranchius furzeri*. This is the shortest-lived vertebrate that can be cultured in captivity, and it replicates many typical phenotypes of vertebrate and human ageing [2, 16–18]. For this reason, it has been used as an experimental model to investigate the effects of several experimental manipulations on ageing [10, 19–25]. Natural habitats of this species can last as short as a month [26, 27] and yet are able to sustain a viable population since sexual maturity can be reached within 2 weeks from hatching [28]. This implies that embryos remain blocked in diapause for several months or even years, as a safe mechanism to prevent species eradication in the case of a drought year. Our personal observations (AC, DRV) show that also in captivity some *N. furzeri* eggs can remain in diapause II for more than a year. *N. furzeri* therefore represent an extreme case of compressed lifespan among annual killifishes and in natural conditions it spends longer time as embryos in diapause than in post-hatch stages.

Molecular studies of killifish embryonic development are scarce. It is known that diapause II is characterized by reduced protein synthesis, cell cycle arrest and remodelling of mitochondrial physiology, and it is controlled by insulin-like growth factor 1 signalling [29–35]. Recently, RNA-seq studies have shown that gene expression patterns during diapause resemble those observed during ageing [36] and vitamin D signalling controls to the choice between direct development and diapause II [37]. While early embryonic development following fertilization has been in part studied [7–9], the characterization of the physiological and molecular events occurring during diapause I has been started to be investigated only recently [35, 38, 39]. Here, to investigate entry and exit form diapause I and II, we created a stable transgenic line in *N. furzeri* that expresses genetically encoded fluorescent reporters for cell cycle phases using the Fluorescence Ubiquitination-based Cell Cycle Indicator (FUCCI) system that exploits the sequential degradation of fluorescent-tagged fragments of the cell cycle regulators Cdt1 and Geminin by the ubiquitin ligases SCF/Cdh1 and APC/C, respectively [40]. The APC/C is activated in late mitosis and stays active throughout G1 phase, while SCF/Skp2 is an APC/C target itself and thus can only become activated in S/G2 and early mitosis. The Azami-Green and Kusabira-Orange coding sequences were fused to ubiquitination signals recognized by the APC/C and SCF/Cdh1, respectively. Because of the mutually exclusive activity of the two E3 ligases, the Cdt1-based sensor is only visible during G1 phase, while the Geminin-based sensor is only detectable during S/G2/M [40]. This tool enabled us to shed light on the cell cycle characteristics of cells during diapause I and to demonstrate that diapause exit is characterized by rapid and synchronous cell cycle reactivation. These characteristics appear unique among vertebrate embryos.

## Results and discussion

**Fig. 1:**
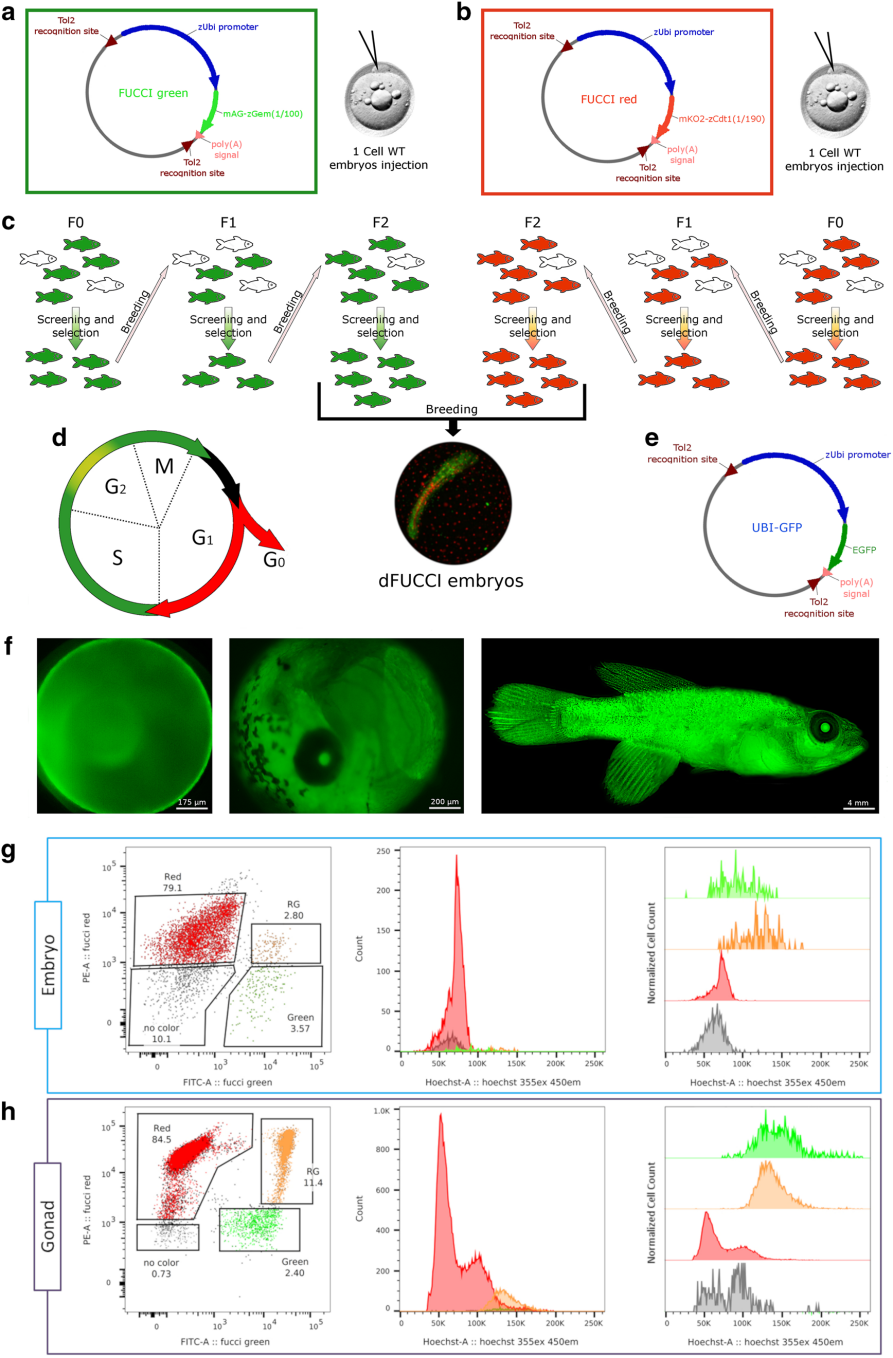
FUCCI transgenic line generation. **a**, **b** Schematic representations of FUCCI green and red constructs, respectively. FUCCI constructs were injected separately in different 1-cell stage fertilized eggs. Positive eggs were raised into adult fish, bred and screened for three generations (**c**). F2 FUCCI green fish were finally bred with F2 FUCCI red fish to generate double FUCCI embryos, which were used for most experiments (**c**). **d** Schematic representation of how FUCCI technology works, cells are green during S/G2/M phases, colourless between M and G1, red in G1 and G0 phases and yellow during a small portion of G2 phase. **e** Schematic representation of zUbiquitin-EGFP construct. EGFP expression driven by zUbiquitin promoter in Nothobranchius embryos and adult fish is shown (**f**). **g** FACS analysis of double FUCCI embryo. The scatterplot on the left shows the gating used to separate the four cell populations. The numbers indicate the percentage of cells in the four populations. The middle graph represents the intensity of the Hoechst staining as measure of DNA content of the four different populations. The graph on the right is the same as the graph in the middle but population are normalized on their own cells count rather than the total cells count. **h** FACS analysis of adult gonads of double FUCCI fish. The scatterplot on the left shows the gating used to separate the four cell populations. The numbers indicate the percentage of cells in the four populations. The middle graph represents the intensity of the Hoechst staining as measure of DNA content of the four different populations. The graph on the right is the same as the graph in the middle but population are normalized on their own cells count rather than the total cells count

To identify a suitable promoter for FUCCI reporter lines, we tested the activity in *N. furzeri* of the zebrafish ubiquitin (ubi) promoter [41] using EGFP as reporter. We observed ubiquitous expression of the EGFP from the second day of development into adulthood in this Tg *ubi:egfp* line (Fig. 1f).

Two different FUCCI transgenic lines were successfully generated using the Tol2 transgenesis system [42–44]: (i) a “FUCCI green” reporter (Azami-Green—Geminin), which is activated during the S/G2/M phase of the cell cycle [40] and (ii) a “FUCCI red” reporter (Kusabira-Orange—Cdt1), which is activated during the G1 phase of the cell cycle [40]. Since the original FUCCI construct is not functional in zebrafish, these constructs were previously modified using sequences from the zebrafish ortholog of Cdt1 and Gemin [40]. We used these constructs without modifications. Both transgenes were placed under the control of the zebrafish *ubi* promoter (Fig. 1a, b), a ubiquitous promoter that drives transcription in all tissues at any developmental stage (Fig. 1e, f).

Adult F0 transgenic fish were screened for fluorescence and bred one to another. F1 fish showing the expected fluorescence pattern were interbred in order to increase the number copies of FUCCI reporter cassettes in their genome, thereby enhancing the fluorescence signal in the F2 generation (Fig. 1c). F2 transgenic fish were used to characterize the expression pattern of the FUCCI reporters at different developmental stages.

To confirm that red fluorescence labels cells in the G0/G1 phase and green fluorescence labels cells in the S/G2/M phase (Fig. 1d), we performed FACS analysis on newly hatched embryos and on adult testis that represent a highly mitotic organ. We could differentiate four types of cells: “black” cells, devoid of fluorescence, red fluorescent cells, green fluorescent cells and double-labelled cells. Analysis of DNA content in these four different types of cells clearly showed that “dark” and red fluorescent cells correspond to G0/G1, green fluorescent cells correspond to S phase and double-labelled cells showed higher DNA content that green fluorescent cells, indicating that they were in G2 phase (Fig. 1g). This phenomenon is expected since, because the APC/C E3 ligase is activated only during late mitosis and, if G2 is of sufficient length, the Cdt1 sensor can accumulate again in G2 phase, resulting in double-labelling of these cells.

We describe the expression pattern of the transgenes in two parts: the first part provides a general description of the fluorescent signal in the single lines (FUCCI green and FUCCI red, respectively) during focal stages of embryonic development and in adult life. The second part is focused on the double transgenic line, where both the red and green signals are present. In this part, changes in relative intensity of the two signals are described and interpreted with respect to the dynamic processes that characterize the different phases of killifish embryonic development.

### Part 1

#### FUCCI red

**Fig. 2:**
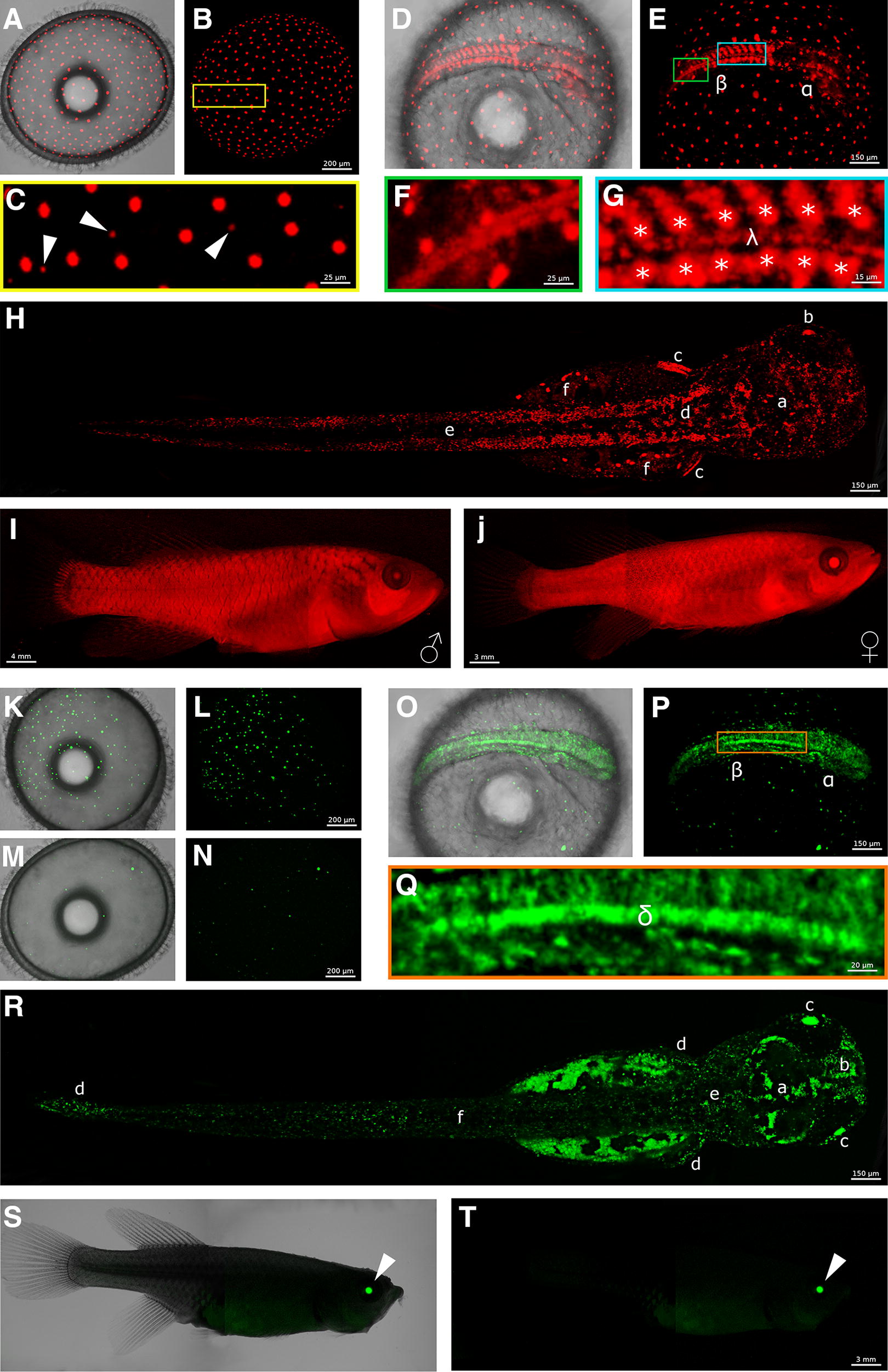
FUCCI green and FUCCI red characterization. **A** to **J** show FUCCI red expression at different developmental stages. **K** to **T** show FUCCI green expression at different developmental stages. **A**–**C**, **K**–**N** dispersed phase. **D**–**G**, **O**–**Q** somitogenesis stage. **H**, **R** hatched fry. **I**, **J**, **S**, **T** adult fish. For a detailed description, see the main text

The FUCCI red signal is localized in the nuclei of cells in every stage of *N. furzeri* life. During the dispersed phase (Fig. 2A, B), two cell types expressed red fluorescence: large cells of the enveloping layer (EVL) and some smaller cells of the epiblast (Fig. 2C, arrows). The nuclei of EVL cells ranged from 22 to 27 μm in diameter and formed a regular array over the yolk surface. The nuclear diameter of epiblast cells was smaller, on the order of 7–9 μm. Both these cell types were red, but the epiblast cells were most likely in G1, since their fluorescence faded and increased over time course of hours, indicating that they were engaged in the cell cycle. By contrast, the EVL cells appeared to be in G0, since the red fluorescence never faded and lasted throughout embryonic development, until hatching.

At the somitogenesis stage (Fig. 2D, E), the regular distribution of the EVL cells remained unchanged. All the other red cells in the embryo showed a patterned distribution that was particularly striking in the embryo trunk, where the somites were clearly delineated (Fig. 2E, β). In the older more rostral somites (Fig. 2G), the inner part of the somites showed a high concentration of red cells, while in the more caudal part of the embryo (Fig. 2F)—where somites were still forming—the red cells were more spread and diffused. Once the new somite pair was completely formed, red cells increased in numbers, becoming more dense and localized in the inner part (Fig. 2G, stars), becoming constitutively red. Just below the notochord midline, another narrow streak of red cells extended from the tip of the head region to the tip of the tail (Fig. 2G, λ). Also, the head region contained several areas with red cells, but these were quite rare, spread out and did not demarcate specific areas (Fig. 2E, α).

Hatched fry had large fraction of red cells (Fig. 2H). The lateral muscles of the trunk (Fig. 2H, d) and of the tail (Fig. 2H, e) harboured a large number of red cells. In the head region, nearly every part of the brain had some spread red cells or red cells aggregates (Fig. 2H, a). The lens (Fig. 2H, b) showed strong red fluorescence at this stage. This could be an artefact due to the high protein stability in this region and lack of degradation of the FUCCI reporter. What remained of the yolk at this stage was still surrounded by large red cells belonging to the EVL, blocked in G0 and not cycling (Fig. 2H, f). Lastly, a large patch of red cells was observed corresponding to the pectoral fins (Fig. 2H, c).

The adult FUCCI red transgenic fish appeared completely red under fluorescence since many cells were in G0 or possibly G1 phase (Fig. 2I, J). Males and females showed a pattern that was virtually identical, and the signal intensity was comparable between different specimens (Fig. 2I, J).

#### FUCCI green

During the dispersed phase, different proportions of green epiblast cells were detected in different FUCCI green embryos belonging to the same clutch of eggs. The number of green nuclei observed varied between 20 (Fig. 2M, N) and 200 (Fig. 2K, L), with a size range between 7 and 25 μm in diameter, typically showing a higher amount of the smaller cells. During this developmental stage, cells arranged randomly over the yolk surface.

In developing embryos, during somitogenesis proliferating green cells were detected in every part of the forming embryos (Fig. 2O, P). The signal was moderately strong in the trunk, in the tail (Fig. 2P, β) and in the head primordia (Fig. 2P, α). The maximum intensity of the signal, i.e. the maximum density of proliferating cells, was limited to a narrow region along the midline, that extended from the end of the head to the end of the tail, between the yolk surface and the lowest part of the somites (Fig. 2Q, orange box, δ). Many green cells migrated over the yolk surface during all of somitogenesis.

In the hatched fry, the proliferative regions in the embryos were more defined. In the torso and the tail, green cells were spread out but homogeneously distributed (Fig. 2R, f). Slightly more dense green cells proliferated in the caudal and pectoral fins (Fig. 2R, d) and at the base of the head in the hindbrain (Fig. 2R, e). The optic tectum (Fig. 2R, a) showed a clear pattern with thick and dense aggregates of green cells at the borders, in the proliferating niches, with almost no green cells in the inner part, appearing completely dark. The olfactory bulb (Fig. 2R, b) in the fry was one of the major proliferating regions, composed by thick streaks of green cells. Lastly, the lens appeared green (Fig. 2R, c), but as for the FUCCI red transgenic line, this could be an artefact due to lack of degradation of the FUCCI reporter. In adults, no green cells could be detected with a stereomicroscope and the only green signal detectable was confined to the region of the eyes (Fig. 2S, T, arrow).

### Part 2

F2 fish were crossed (FUCCI red with FUCCI green), generating double FUCCI green/red embryos (dFUCCI), which were analysed by means of time lapse confocal imaging. Embryos from this cross were imaged for periods spanning from hours to days at different stages of development from the end of epiboly to late somitogenesis, i.e. past the stage when embryos entered diapause II. The stacks of images of each time point were then processed with IMARIS software to perform particle tracking and counting.

These experiments required occupancy of the setup for considerable amounts of time, as every single time lapse acquisition lasted from 8 h to 4 days, limiting the number of replicates available for each stage (Table 1). This study was designed to provide an overview, as complete as possible, of *N. furzeri* embryonic development (compromising on the number of replicates) as opposed to deep analysis of only one specific stage (e.g. reaggregation) with a larger number of replicates. Because the morphology of South American and African annual killifishes is comparable, we follow here the staging developed by Wourms for the South American annual killifish *Austrofundulus limnaeus* [6] for reference.

**Table 1:** Overview of dFUCCI embryos imaged for each developmental stage

Embryonic stage	Time required to reach the stage in *N. furzeri* at 28 °C	Stage duration at 28 °C	Amount of embryos imaged
Dispersed phase (WS 19–20)			
Early (small number of green cells)	4 days	1 to 30 days	4
Late (larger number of green cells)	7 days on average	12–24 h	6
Dispersed phase transition (from few to many green cells)	5–7 days on average	2–3 days	4
Reaggregation phase (WS 21–25)	8 days on average	8–12 h	4
Extension phase (WS 26)	9 days on average	8–12 h	4
Somitogenesis (without diapause II arrest) (WS 29–33)	10 days on average	5–8 days	6
Diapause II arrest and release	11 days on average	Days to years	4

It shows the number of embryos acquired at each developmental stage. From stage to stage, embryos acquired could be the same, acquired progressively during its development, or different ones. WS means Wourms’ stage [12]

In his work, Wourms described the stages of killifish development from egg fertilization to fry. He dived the development in a total of 46 stages: 43 pre-hatching and three post-hatching. Stage 1 defines the freshly fertilized 1 cell stage embryo, while stage 46 describes the fry after digestion of the yolk that is starting to hunt prey.

In our work, we focus on a subset of these stages and, more precisely, we describe development from stage 19 to 33, which correspond to the phase between completion of epiboly and beginning of the dispersed phase to an advanced stage of somitogenesis.

### End of epiboly (WS 19)

**Fig. 3:**
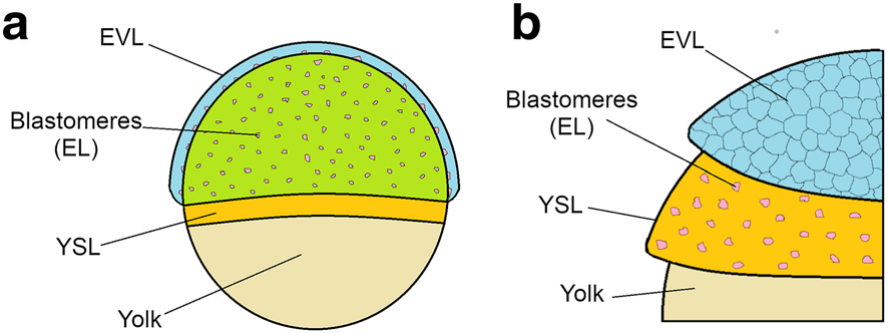
Schematic representation of embryonic layers in annual killifish species. Yolk syncytial layer (YSL) is made of cell forming a syncytium and in direct contact with the yolk. On top of this layer migrate and divide the blastomeres, a discrete population of cell composing the epiblast layer. The enveloping layer (EVL) is the layer of cells on top, a thin layer that completely envelope the other two. Two representations are shown, a lateral semi-transparent view (**a**) and a cutaway representation (**b**)

Detection of fluorescence signal with a confocal microscope was not possible before the stage of 70% epiboly because the expression of the fluorescent reporters was too weak: earlier stages of epiboly were described previously by injecting synthetic RNA coding for FUCCI reporters [7]. During epiboly, three cell layers become defined: the yolk syncytial layer (YSL), the enveloping layer (EVL) and the epiblast layer (EL) (Fig. 3).

Two of these three cell types could be clearly distinguished based on dFUCCI signal: the EVL cells and the EL cells, which could be distinguished based on their non-overlapping spans of nuclear sizes (21–27 μm vs. 8–12 μm, respectively). The EL cells (showing either green or red fluorescence) migrated in an apparently random direction in the space between the YSL and the EVL. Remarkably, these cells continued their movements also once epiboly was completed. Random movements of EL cells in the dispersed phase were originally reported by Lesseps et al., in the 1970s [45] by means of bright-field microscopy and were later confirmed by us [6].

**Fig. 4:**
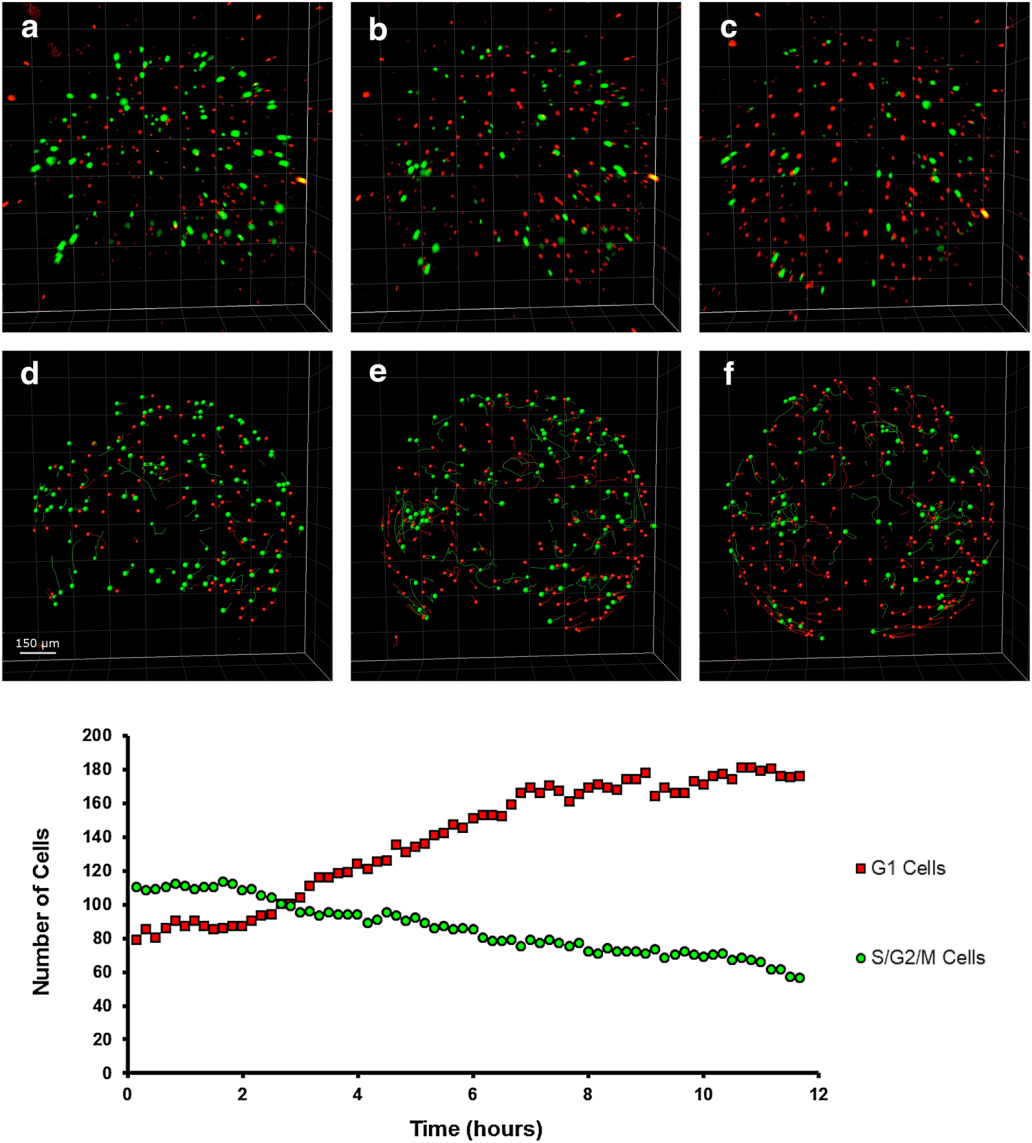
Cell dynamics during epiboly (Wourms’ stages 18–19). When epiboly is ongoing, both green and red cells are present in dFUCCI embryos (**a**). As long as epiboly proceeds (**b**, **c**) the number of green EL cells gradually and slowly decreases over time, while the number of red EVL cells increases until the reaching of a plateau (graph hours 7–12). The cells in the field of view were easily tracked and counted transforming the dots in particles with IMARIS (**d**–**f**). The images and graph refer to the acquired portion of the embryos, corresponding to the superior hemisphere

EVL cells maintained red fluorescence for the entire span of subsequent development, possibly indicating arrest in G_0_ phase, which is coherent with the not proliferating status of these cells. Additionally, their number remained stable around 200 in the portion of the embryo that could be imaged (corresponding roughly to the superior pole) (Fig. 4). EVL cells showed a directional movement until the completion of epiboly (Wourms’ stages 18–19) (Additional file 1: Movie S1), when they reached their final position and constituted a syncytium, which was then maintained during the ensuing development. This physical movement and positioning of EVL cells play an important role during the development of killifish embryos. The spreading of EL cells is indeed instructed by mechanical interactions between EVL and EL cells [8].

**Fig. 5:**
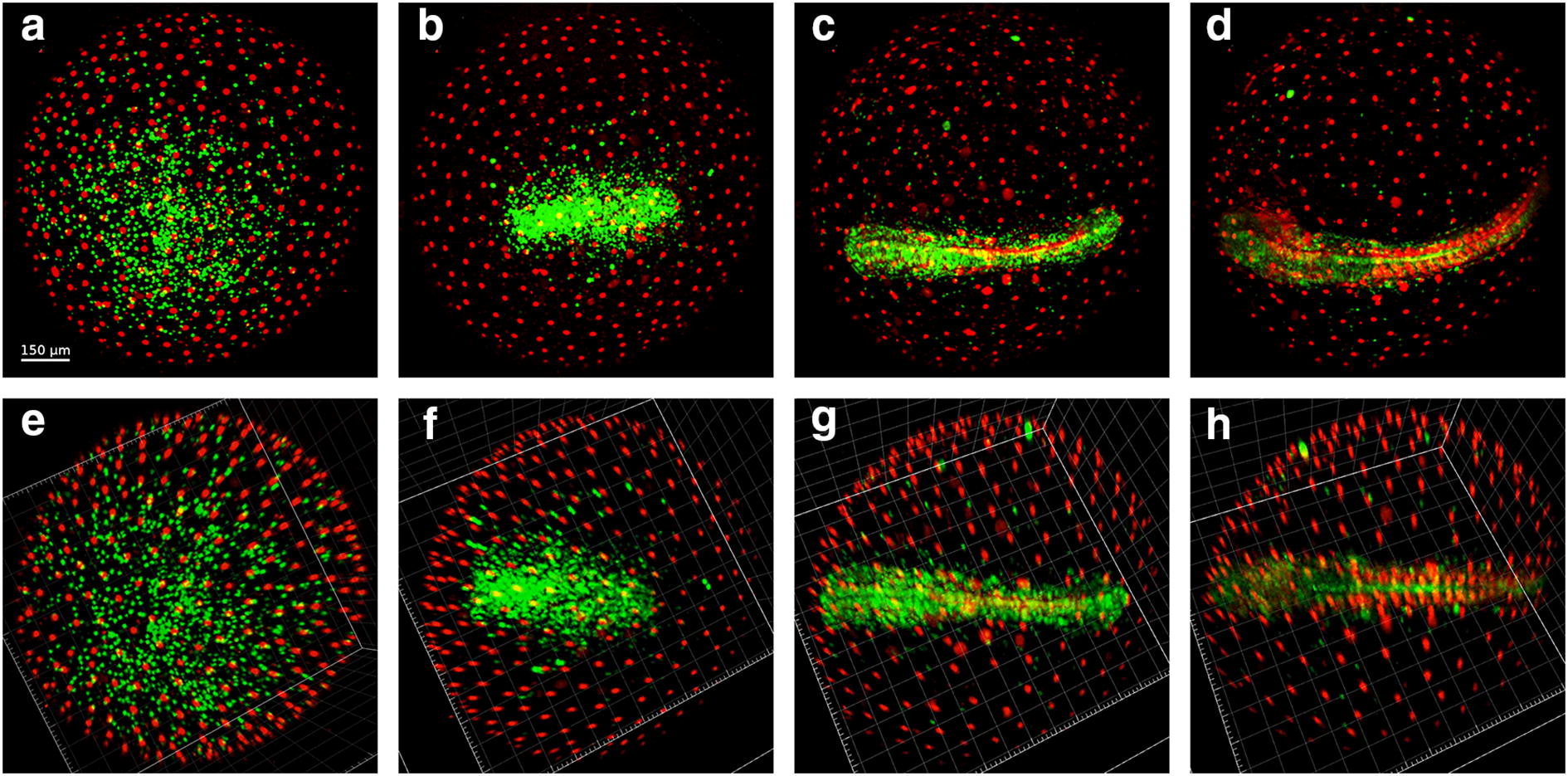
ESL nuclei as reference point for drift correction. Embryos continuously move throughout development (**a**–**d**), but EVL nuclei do not move once they reach their final position at the end of epiboly. These nuclei can therefore be used as reference system and drifts and rotations that occur during development can be corrected using their position (**e**–**h**). Correcting the drifts allows a more precise and reliable cell tracking and data analysis

EVL cells formed a defined and regular architecture, tiling the entire surface at an average distance of about 80 μm from each other. Therefore, we used the position of these nuclei as a reference to correct for yolk movements that often occurred in the embryos during development, allowing for precise tracking of the movements of all the other cells and developing structures (Fig. 5).

### Early dispersed phase (Wourms’ stages 19–20) and diapause I

**Fig. 6:**
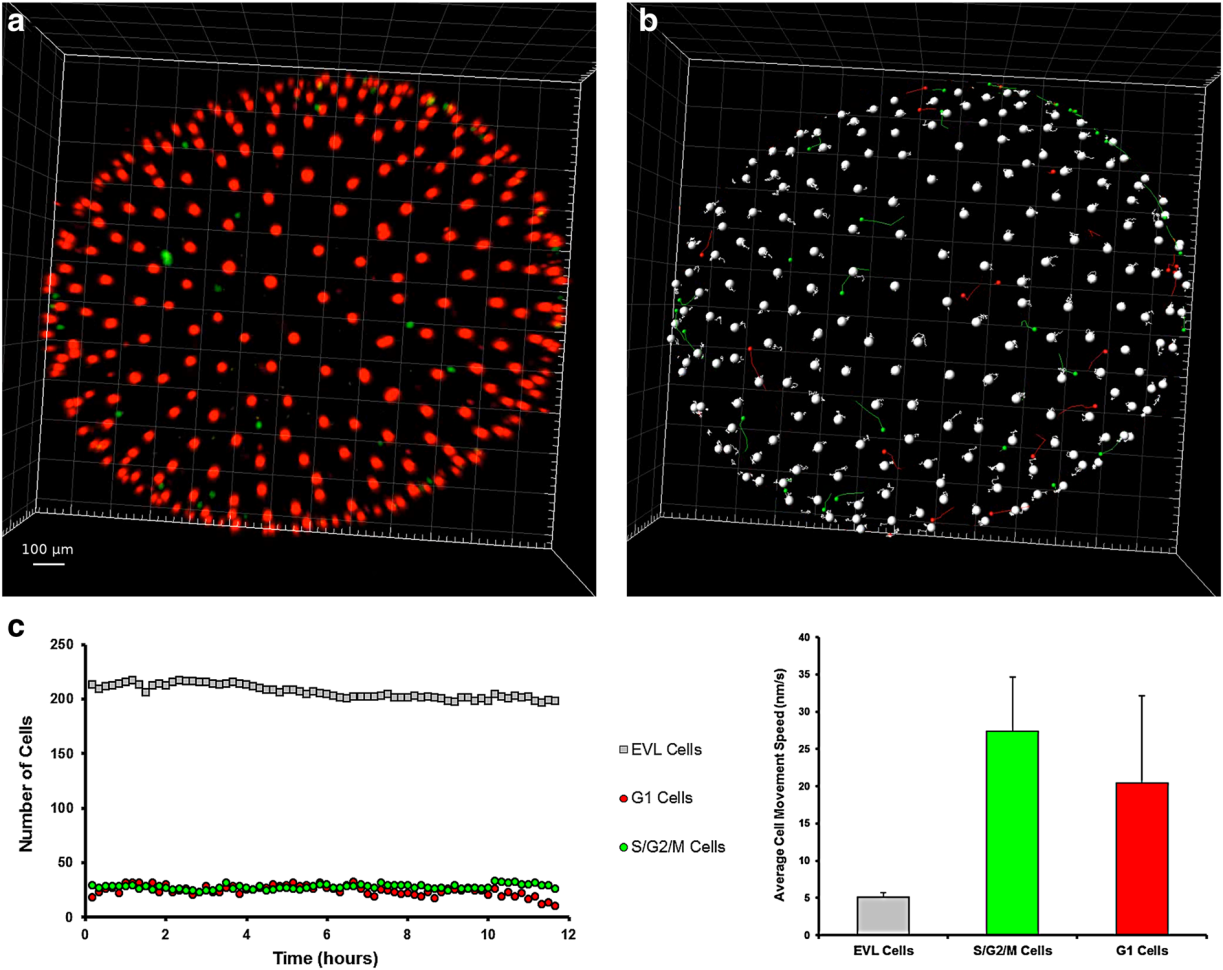
Cell dynamics during the early dispersed phase (Wourms’ stages 19–20). The first part of the dispersed phase is characterized by the presence of few EL green or red cells (**a**). ESL cells are pseudocoloured as large white dots and EL green and red cells as small green and red dots, respectively (**b**). Tracking of the three cell types over time. The number of each type of cell was constant for more than 10 h (**c**) and EL cells continuously moved during the whole time. Tracks of individual cells are shown in **b**, and speed average values in **c**, right panel. The images and graph refer to the acquired portion of the embryos, equivalent to the superior hemisphere

When epiboly ends and the dispersed phase begins, the number of detectable green and red EL cells was reduced from more than 200 to less than 70, in a ratio of almost 1:1 (Figs. 4 and 6c, left panel). These cells are actively moving in a seemingly random fashion (Fig. 6b, c, right panel). It is important to remark that the decline in green and red cells does not correspond to cell loss, as EL cells are clearly detected by bright-field microscopy at this stage (Additional file 2: Movie S2, min 0.53 to 1.10). Previous studies of early cleavages by means of injection of synthetic FUCCI mRNA [7] also reported a “dark phase” when neither red nor green fluorescence is detectable. The majority of EL cells appear to be locked in this “dark phase” during early dispersed stage. Based on FACS analysis of embryos in later stages of development (Fig. 1g, h), we should assume that these “invisible” cells are in G1 phase. A G1 block is indeed observed also in the South American killifish *A. limnaeus* [32].

In addition, all the embryos we could observe at the end of epiboly (*N* > 30, including embryos that were not followed in time lapse but only through still imaging) transited through a phase when only few EL green or red cells were detectable, characterized by the following features:

- Regularly spaced EVL large cells nuclei (19–25 μm nucleus diameter) that do not move, migrate or divide (Fig. 6).
- Presence of few (less than 80) red and green small epiblast cells (7–13 μm nuclear diameter) that do not divide (Fig. 6).
- Seemingly random cell movements, along with “dark” cells visible only in bright field (Additional file 2: Movie S2).

Early dispersal occurred in all embryos and was reached by a continuous decrease in green cells and an increase in red cells during epiboly (Fig. 4), leading finally to a stabilization of cells number (Fig. 6c). It has been recognized already in the 1970s that duration of early dispersal is variable (from hours to days); indeed, this stage corresponds to diapause 1 (DI) [4]. Noteworthy, DI is characterized by a complete stop of the cell cycle with cell possibly synchronized in G1 phase. However, DI is far from being a static stage, as cells constantly move over the yolk surface (Additional file 2: Movie S2, min 0.53 to 1.10). The functional relevance of erratic cell movements remains, for the moment, unexplored.

### Synchronized cell cycle re-entry underlies release from diapause I

**Fig. 7:**
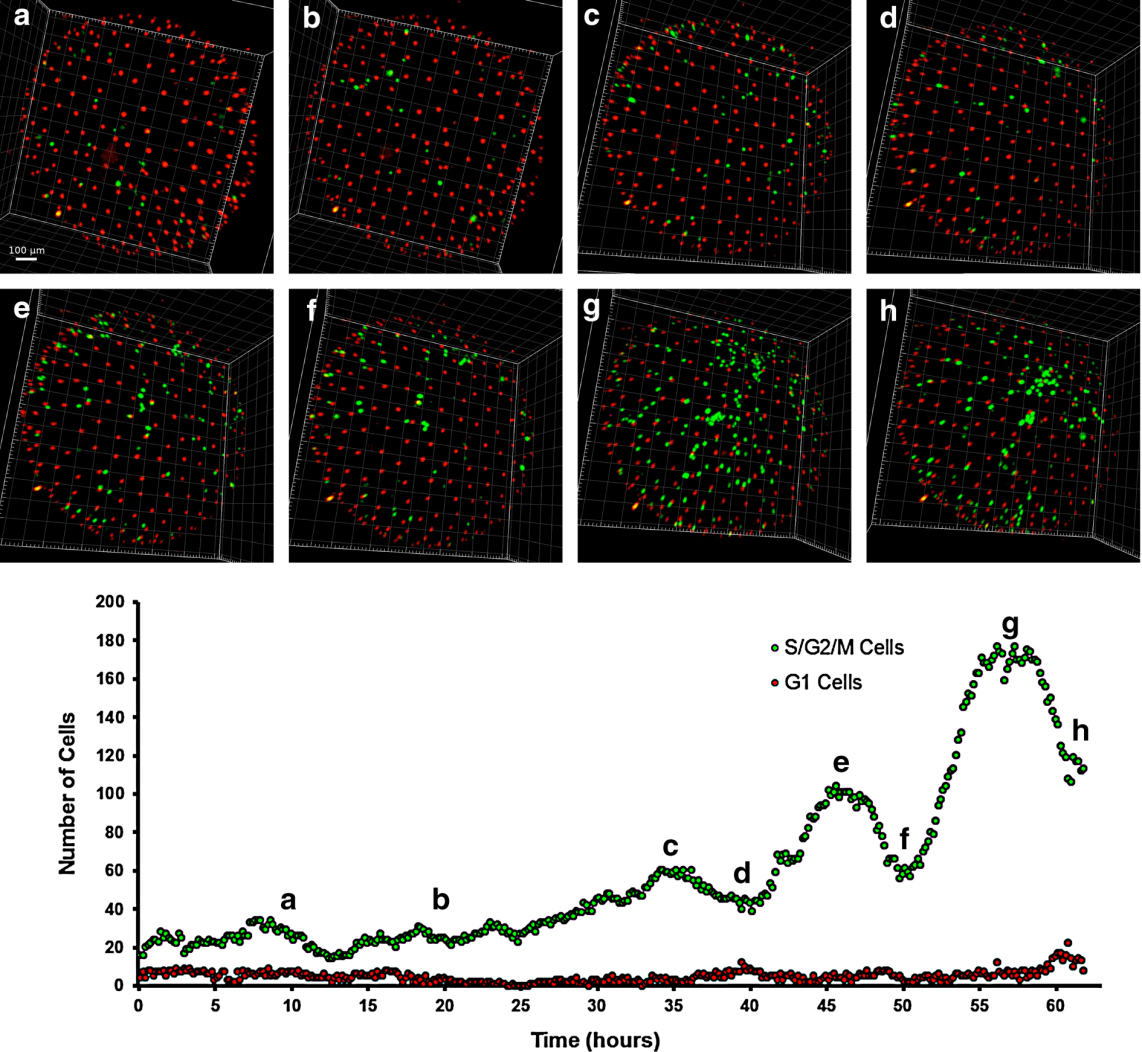
Transition from early to late dispersed phase. **a**–**h** Time lapse showing the transition from a stage with few EL red or green cells could be detected through multiple reactivation and division events up to a relatively stable condition where the number of green EL cells is ~ 5 times their original number. **g** Quantification of green EL cell numbers over time. The letters **a**–**h** correspond to the pictures shown in **a**–**h**. Multiple peaks of synchronized proliferation are clearly visible (**c**, **e**, **g**) and divided by phases when cells synchronously enter into G1 phase (**d**, **f**, **h**). The images and graph refer to the acquired portion of the embryos, equivalent to the superior hemisphere

The mechanisms responsible for release from diapause I are unknown, although previous experiments suggest that it is a temperature-dependent process [14, 46, 47]. The majority of embryos imaged during dispersed phase were either in a phase with few green cells (80 or less) or in a phase with a larger number of green cells (300 or more), indicating that the duration of the transition is considerably shorter than either of these two phases and difficult to capture. We were nonetheless able to image the exit from DI in four embryos. In all four cases, the release from DI was characterized by the rapid appearance of a large number of green fluorescent cells that was not preceded by a rise in red cells, indicating that: (i) the “invisible” EL cells were indeed synchronized and (ii) most of the “invisible” EL cells, where both reporters are actively degraded, entered into S/G_2_/M phase without previous accumulation of the red reporter (Fig. 7). This indicates that either the transition is rapid or the regulation of E3 ligases during diapause I differs from their regulation during the normal cell cycle. Remarkably, these reactivated cells showed what seemed to be a pattern of synchronous division, generating a peak of green cells followed by a period of reduced cell numbers (likely because some cells entered in the “dark” phase). In two out of the four imaged embryos, subsequent pulses with a periodicity of 10 h were apparent. Cells alternated from a green phase to a dark phase without showing a red phase, a pattern typical of early cleavage in *N. furzeri* [7]. Peaks of green fluorescent EL cells were present, but less apparent, in a third embryo whereas only the first peak was clearly visible in the fourth embryo (Additional file 3). This synchronization may simply reflect the fact that cells are synchronized during G1 and also cell cycle exit is synchronized. This is, however, a totally unique feature of embryonic development as cell cycle synchronization is otherwise known only for the very early phases of development that are controlled by maternal transcripts.

In all four cases, the number of cells increased over time—indicating active division—and the stage with ~ 300 green cells was reached in about 36 h. In addition, the proliferating cells moved erratically during the reactivation, in a way comparable to the movements of epiblast cells that were documented during DI by bright-field microscopy (Additional file 2) or during early dispersed phase by confocal microscopy (Fig. 4).

### Late dispersed phase (Wourms stage 20)

**Fig. 8:**
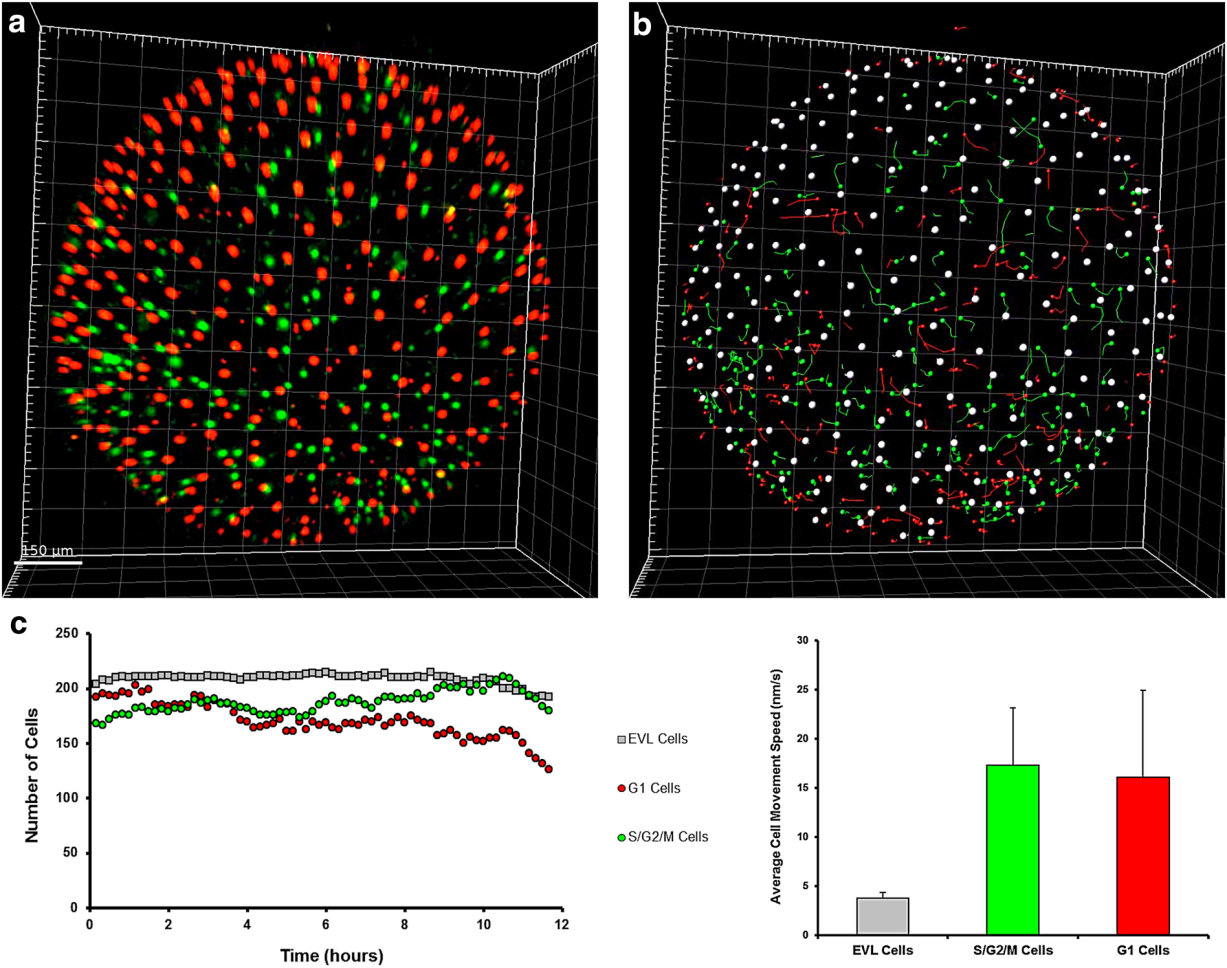
Cell dynamics during late dispersed phase (Wourms’ stage 20). The second part of the dispersed phase is characterized by the presence of a larger number of EL green or red cells as opposed to early dispersed phase (Fig. 4). (**a**, **b**) Cell tracking. EVL cells are visualized as large white dots, while EL green and red cells as small green and red dots, respectively. All detected cells were tracked over time. **c** Quantification of the numbers of each type of cell over time. Note that the number is relatively constant for more than 10 h, while still moving. The images and graph refer to the acquired portion of the embryos, equivalent to the top superior hemisphere

The late dispersed phase (Fig. 8) was characterized by an almost equal number (~ 500) of EVL, EL green and EL red cells. Random movements of EL cells continued at a speed comparable to that observed during the early dispersed phase and diapause I (Fig. 6b, c, right panel). The detectable EL cells did not increase in numbers. The amount of time embryos spent in this second part of the dispersed phase could not be determined. At the moment, we cannot offer an explanation for this remarkable phenomenon.

### Reaggregation (Wourms’ stages 21–26)

**Fig. 9:**
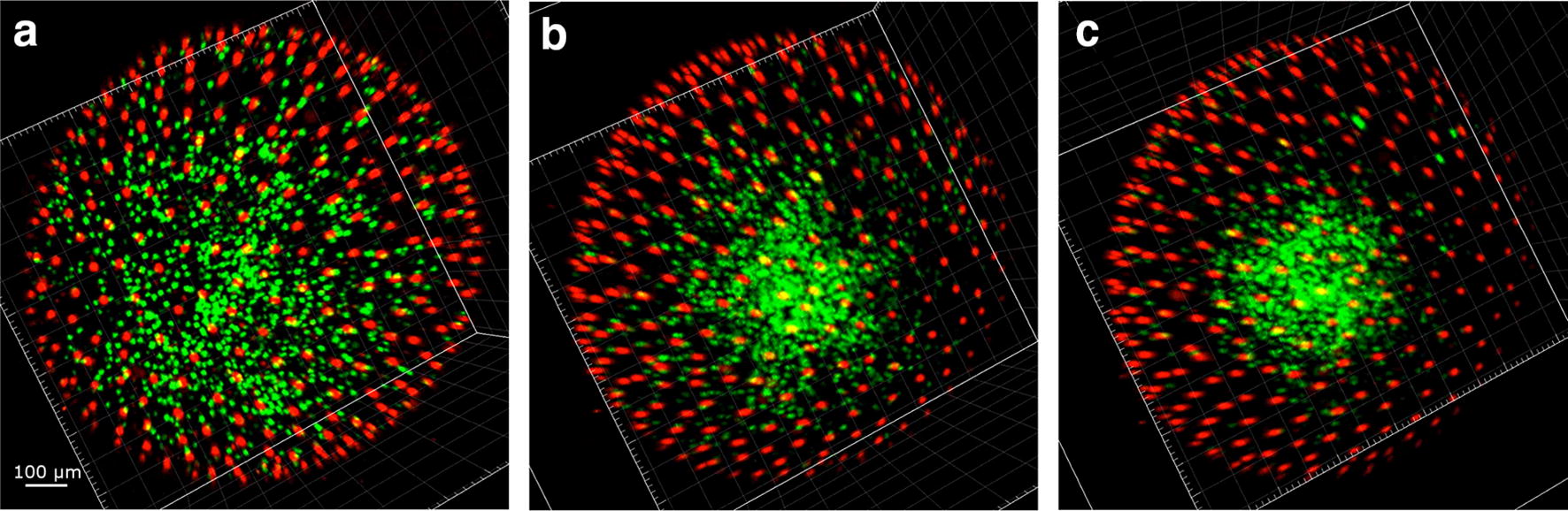
Reaggregation phase (Wourms’ stages 21–25). Green EL cells converge initially onto a single region of the embryo (**a**–**c**), forming a circular structure that over time becomes more compact with a reduced radius

Reaggregation starts when the majority of EL cells change their movements from erratic to directed towards a specific region of the embryo, forming an initially sparse circular aggregate, whose radius becomes progressively smaller (Fig. 9 and Additional file 4: Movie S3 min 0.00 to 0.12). It was not possible to determine the point of the embryo towards which cells migrated in our experiments, but experiments performed by Wourms in 1972 [6] suggest that this is located in the lower hemisphere of the egg. The movements of the cells in this region were greatly reduced and the circular formation reduced its diameter over time. The reaggregation process required a small fraction of total developmental time, and in about 15 h the final circular aggregate of green cells was completely defined. A more extensive description of the aggregation and gastrulation processes in annual killifish and the associated cellular movements was recently provided by Pereiro et al. [9] and is beyond the scope of the present study.

**Fig. 10:**
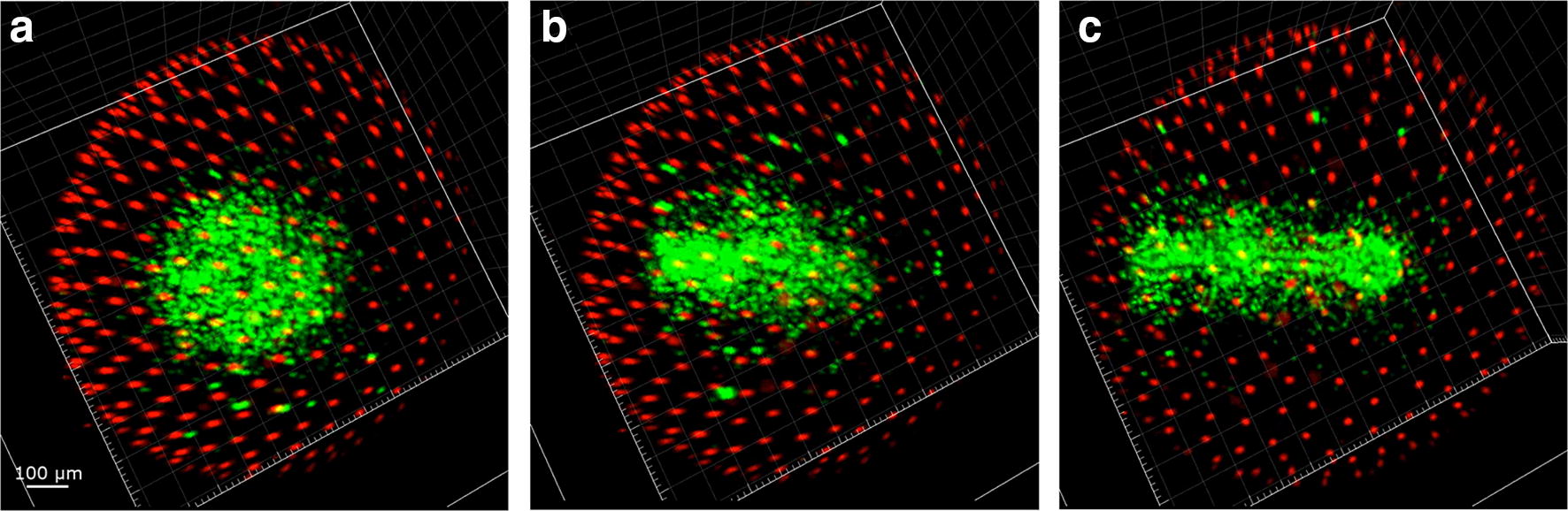
Axis extension phase (Wourms’ stage 26). The initial circular formation (**a**) lengthens to form the primordial axis (**b**, **c**)

The circular organization progressively changes in shape without varying its area, becoming an ellipsoid that extends along its main axis retaining a higher density of green cells in the inner part and a decreasing density on the borders (Fig. 10 and Additional file 4: Movie S3 min 0.12 to 0.21). Of note, the embryo is formed mainly by green cells with very little fraction of red cells, possibly because cell multiplication is necessary to reach a sufficient cell number for all the different embryonic structures to be formed and so the predominant cycle phases are the proliferation phase S/G_2_/M. In this phase, the number of epiblast green or red cells that do not belong to the embryo primordium and show erratic movements appears to be greatly reduced and seems comparable or smaller than the number of cells that randomly move during diapause I (< 80).

### Somitogenesis (Wourms’ stages 29–33+)

The description of somitogenesis refers to direct-developing (DD) embryos. We were unable to image any diapause-committed (DC) embryo during this phase, most likely because the temperatures reached in the imaging chamber (> 26 °C) prevented diapause induction.

**Fig. 11:**
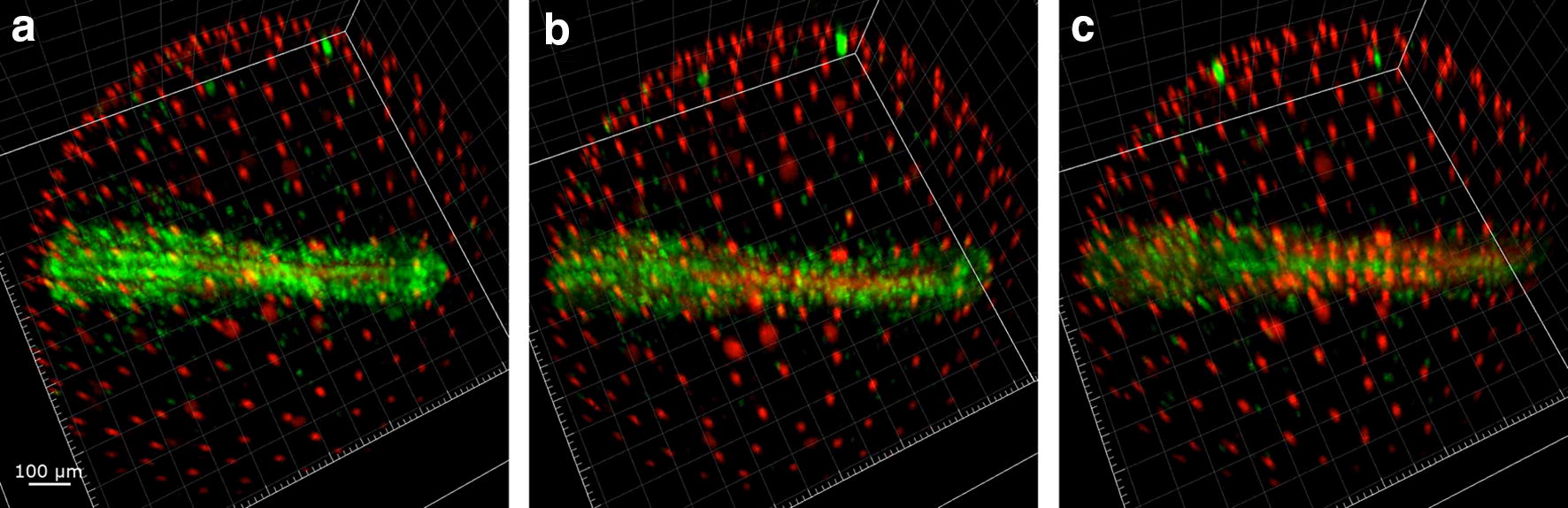
Direct-developing embryos late somitogenesis (Wourms’ stages 29–32). Green cells populate the whole embryonic axis for all the somitogenesis in embryos that do not enter diapause II. Three subsequent stages of the same embryo are reproduced to illustrate how green cells number and density slowly drops as long as development proceeds. **a** The embryo is composed mainly of green cells, **b** red cells increased with time and populated the first the somite pairs **c** somites contain red cells along almost the entire antero-posterior axis of the embryo

Somitogenesis is associated with changes in expression of the FUCCI reporters that are highly reminiscent of those first described during zebrafish development [40]. The somites are the first structures where red fluorescence predominates. Their formation is progressive and is completed within a few hours (Additional File 4: Movie S3 min 0.26 to 0.52). Somites increase in number by addition of progressively more caudal somite pairs, as typical for teleost embryos (Fig. 11).

After the formation of the first 2 somites, 4 symmetrical green streaks of proliferating cells become apparent, reaching from the caudal margin of the head to the end of the tail: two inner proliferation streaks, close to the midline and divided by the midline itself, and two outer streaks, defining the outer borders of the embryo (Additional file 5). The somite structures are located between the inner and outer streaks of green cells.

The two inner streaks contain cells that migrate inwards (Additional file 5: Figure S2), while the movements of the cells of the outer streaks do not follow a clear direction. Some cells belonging to the outer streaks migrate outwards.

As somitogenesis proceeds, the green signal slowly reduces its intensity due to the reduction in the fraction of proliferating cells. Somites, that are composed mainly of red cells, are progressively added posteriorly determining the growth of the embryo in the longitudinal dimension (Additional file 5, min 0.40 to 0.53). However, even at late stages of somitogenesis, DD embryos are never exclusively dominated by red cells, which would be expected in case of a complete cessation of proliferation events, as for embryos in DII (Fig. 13).

### Diapause II

**Fig. 12:**
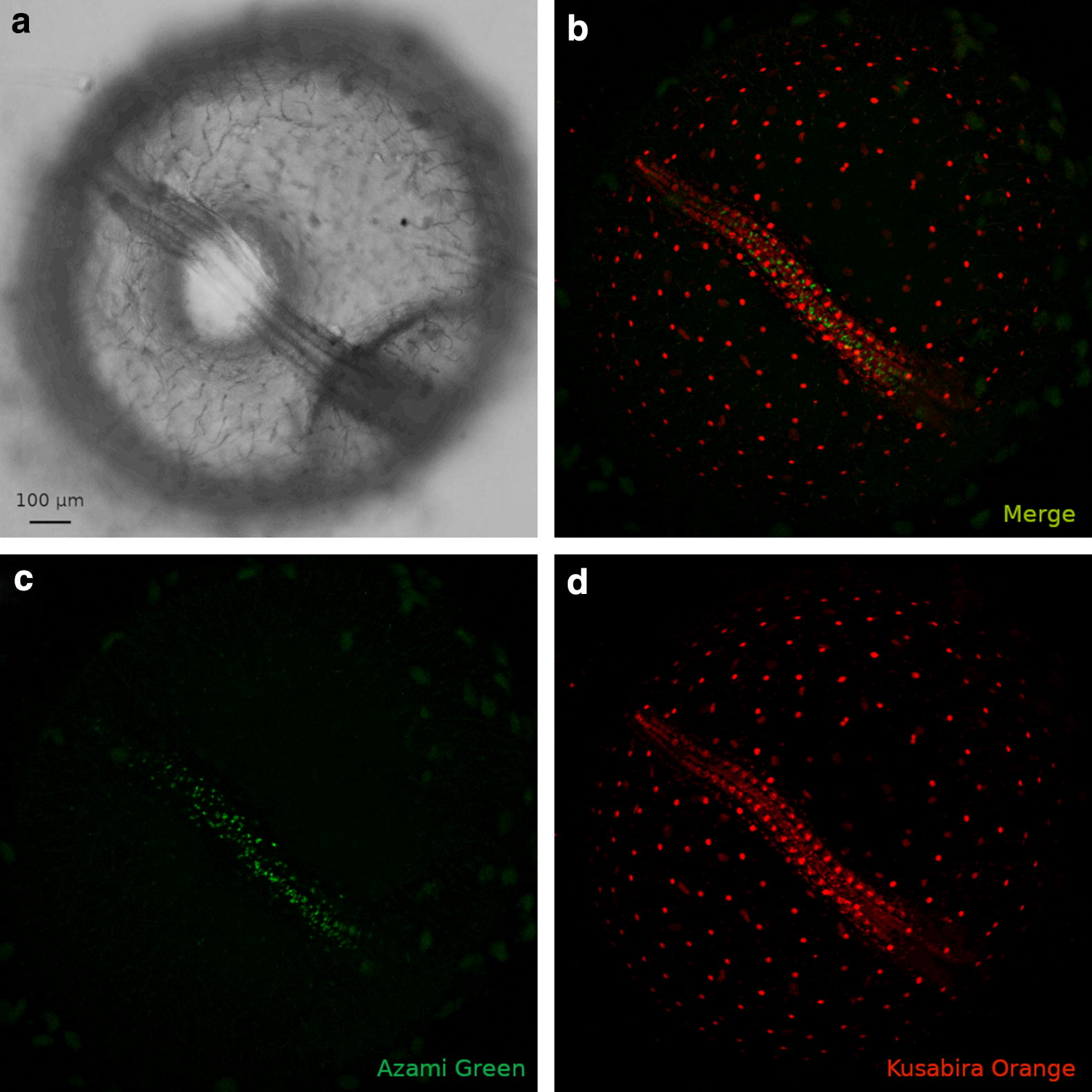
Diapause II arrested embryo. An embryos arrested in diapause II is illustrated in brightfield in (**a**). **b** Overlay of green and red signal. **c** Green cells are concentrated in the medial part of the embryo between the somites. **d** Red cells clearly define the already formed somites and diffusely populate the whole axis region

**Fig. 13:**
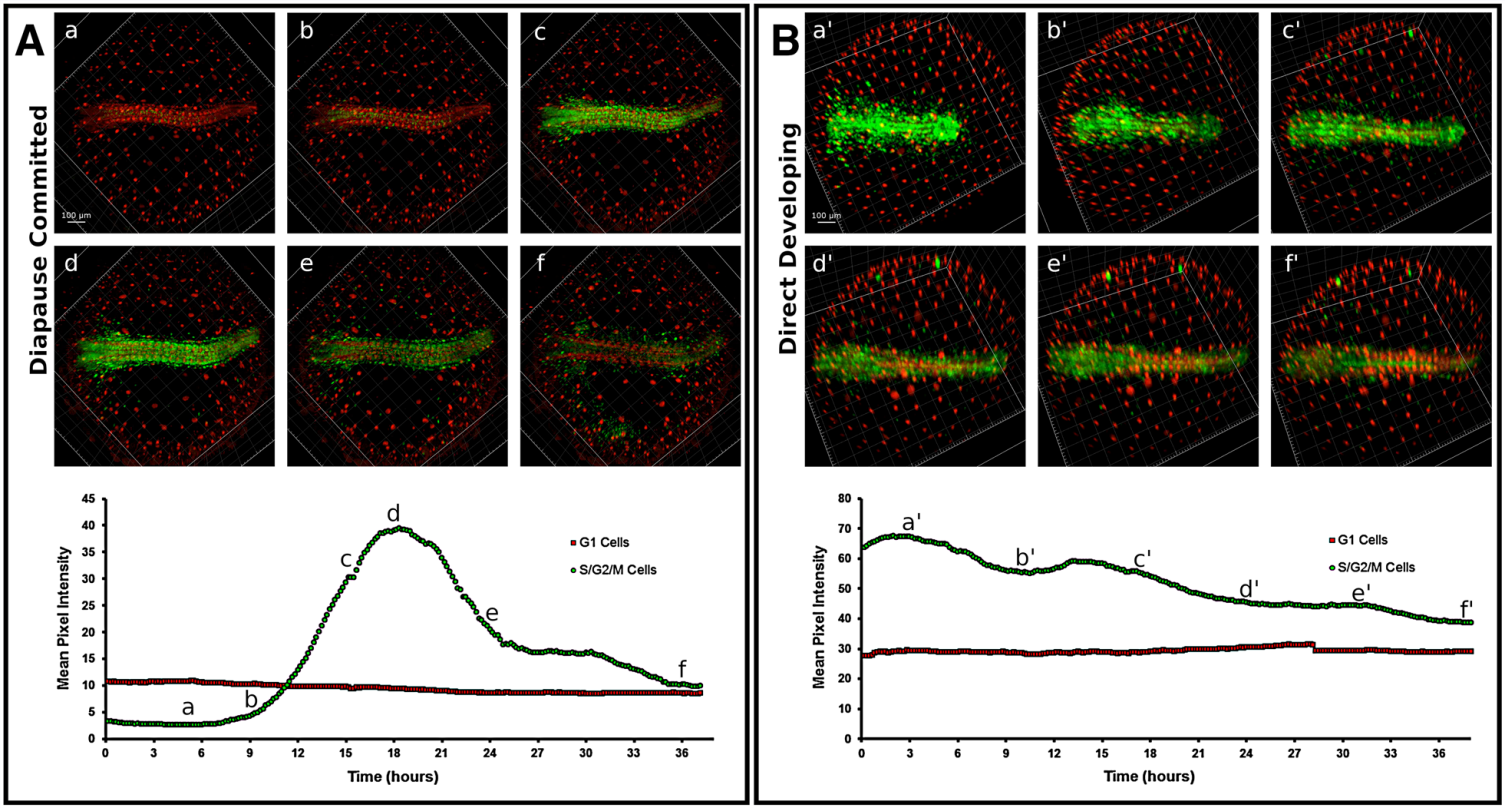
Time lapse of red and green fluorescence. Release from diapause II (**A**) as opposed to direct development (**B**). Upon release from diapause II, green cells greatly increase their number and density in less than 10 h (**A**). After the initial proliferation burst (c, d), green cells slowly decrease in number and density (e–f). In the lower panel, a quantification of green signal intensity as an indirect estimate of the number of green cells is reported. The lettering indicates the correspondence of the curve with the pictures (a–f). **B** An example of direct development. The number of green cells decreases gradually, while the number of red cells is roughly constant (a’–f’). Note that at the end of the processes the direct developed embryo (f’) is very similar to the embryo that escaped diapause (f). In the lower panel, a quantification of green signal intensity as an indirect estimate of the number of green cells is reported. The lettering indicates the correspondence of the curve with the pictures (a’–f’). Note that at the end of the processes, the ratio between red fluorescence and green fluorescence in the direct developed embryo (f’ in the right panel) is similar to that of the embryo that escaped diapause (f in the left panel)

In order to obtain images from embryos in DII, these were incubated for weeks at low temperature (22 °C) before imaging. At the beginning of the imaging, diapausing embryos contained almost exclusively cells with red fluorescence, indicating an almost complete suppression of proliferation. Sparse green cells could be detected along the midline and spread between the somites, but their number was greatly reduced compared to DD embryos. In addition, these remaining green cells were possibly blocked in G_2_ and not dividing, since the green signal did not increase (Figs. 12, 13A from 0 to 6 h, Additional file 6 from 0.00 to 0.07). For the technical reasons delineated above, it was not possible to document diapause entry, and therefore, it remains unclear whether cell cycle arrest in embryos committed to diapause (DC) is progressive or it is reached abruptly at a specific developmental stage.

### Synchronous cell cycle re-entry and catch-up growth upon release from diapause II

Previous studies in the South American annual fish *A. limnaeus* suggested that exit from diapause is associated with catch-up growth [13]. However, time-resolved studies are missing and the cell cycle status during this process is unknown. The release from diapause II was documented in a total of four embryos (Additional file 6: Movie S4 and Additional file 7: Movie S5), and in all cases we could document a rapid catch-up process by which almost all the previously red or colourless cells of the embryo, with the exclusion of the cells forming the somites, switched to green fluorescence, showing a reactivation of proliferation (Fig. 13A and Additional files 6: Movie S4). Cell cycle reactivation starts apparently simultaneously in the whole embryo without an antero-posterior gradient and is a rapid process with green signal doubling in less than 4 h and reaching a peak in about 10 h (Fig. 13A b–d, Additional file 8). As in the case of release from DI, the upregulation of green fluorescence is not followed by an increase in red fluorescence, suggesting that cells proceed through S/G_2_/M phases without long permanence in G_1_. This burst is transient; once the peak is reached, the green signal halves within about five hours and reaches a steady state within one day (Fig. 13A, d–f, Additional file 8). Our analysis confirms that DC and DD are distinct developmental pathways. Indeed, green signal is reduced but always present in DD embryos, while diapausing embryos show almost total absence of green cells. Another difference between DC and DD is the smaller axis thickness of DC embryos that was already described by Furness et al. [15]. The reactivation results primarily in a widening of the embryo with little longitudinal growth.

The rapid change in morphology in somites and embryos upon reactivation is compatible with the observation that embryos in diapause upregulate genes responsible for protein synthesis, such as ribosomal proteins and initiation/elongation factors [36], despite protein synthesis being suppressed in diapausing embryos [34]. Diapausing embryos could be “primed” to rapidly recover growth, as translation could be more rapid and efficient in re-entering the cell cycle and supporting the metabolic needs of a growing embryo.

## Conclusions

**Fig. 14:**
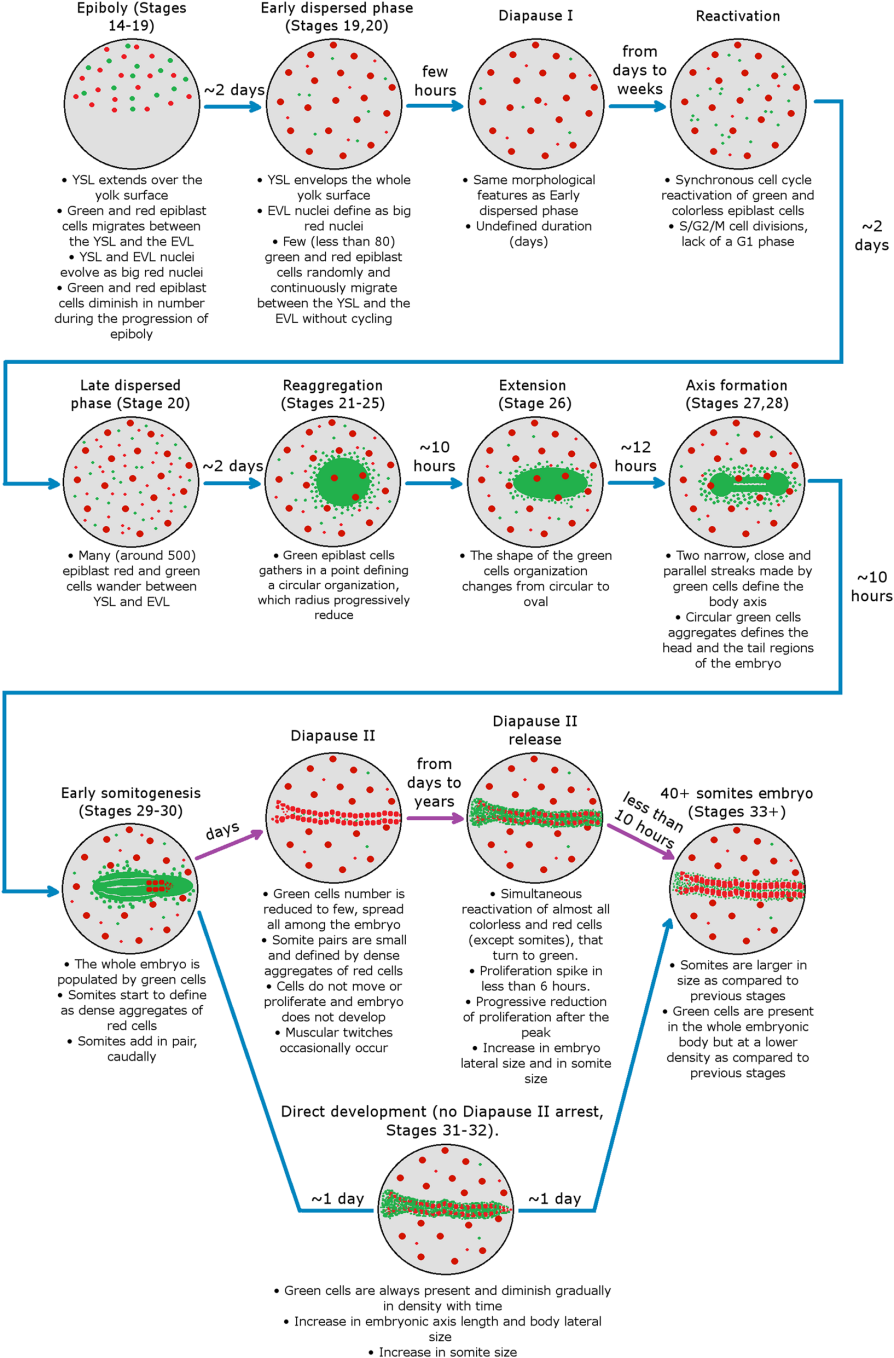
Graphical abstract representing FUCCI *N. furzeri* fish development. Images and developmental times refer to 26 °C incubation conditions, except purple arrows that refer to low temperature (18–21 °C) incubation conditions. Stages refers to developmental stages described by Wourms [6]

Our study improves the existing knowledge on annual killifish embryology and defines the following different phases of *N. furzeri* development based on cell cycle properties that are summarized in Fig. 14. Of particular interest are the following points:

- An early dispersed phase is characterized by cells possibly locked in G1 phase;
- Diapause I is a prolongation of the early dispersed phase when cells randomly move but do not divide;
- Exit from diapause I is characterized by synchronized bursts of cell division;
- After exit from diapause I, embryos do not proceed immediately to reaggregation, but transit through a late dispersed phase with random migration of epiblast cells without cell division
- Exit from diapause II is characterized by a transient burst of cell division and a catch-up process that leads primarily to transversal growth of the embryo with little longitudinal growth.

In addition to the new knowledge we have derived on cell dynamics during embryonic development, we imagine that the FUCCI transgenic line may represent a resource with several applications.

In a first instance, it allows a very precise identification of the timepoint when an embryo exits from DII.

Release from DII is not synchronous, possibly as a strategy of bet hedging [10]. Currently, embryos that exit DII can be only identified based on embryo width, eye size or pigmentation by daily observations. This technique can reliably identify only embryos that left DII a few days in the past and became apparently different from diapausing embryos. With our tool, the time window between DII exit and expression of a detectable signal is narrowed to few hours. This transgenic line, combined with FACS sorting and transcriptomics/proteomics, could offer a new and unique entry point to dissect the molecular mechanisms responsible for exit from quiescence.

A second perspective, that goes beyond developmental dynamics, is the study of cell dynamics in adult organs. In particular, *N. furzeri* represents a convenient model to study the effect of ageing on adult stem cells [48] and tail regeneration [49]. FUCCI offers a direct tool to quantify changes of cell cycle dynamics during aging and in response to regeneration and to purify and analyse adult stem cells/progenitors.

Finally, *N. furzeri* is characterized by a high incidence of age-dependent spontaneous tumours. FUCCI would provide a tool to visualized and investigate tumours in vivo by intravital microscopy [50], allowing longitudinal studies of spontaneous tumorigenesis in living fish, characterization of tumour response to pharmacological treatments or other interventions and sorting of tumour-derived cells for molecular analysis.

## Methods

### Fish maintenance

All adult fish used were raised singularly in 3-L tanks from the second week of life (breeders were kept differently, section below).

Water parameters were ph: 7–8; Kh: 3–5; T: 24–26 °C.

25 to 50% of the water in each tank was replaced with fresh tap water during every week.

Fishes were raised in 12 h of light and 12 of darkness.

Fish were fed with chironomus 2–3 times a day and “Premium Artemia Coppens^®^” 1–2 times a day.

### Breeding

Breeders were kept in tanks of a variable size from 8 L size to 130 L size, at an average density of 1 fish every 2 L (capped at 35–40 fish total for the biggest tanks) and with a male/female ratio of 1:3.

To start the breeding event, one or more boxes (variable size from 20 cm × 15 cm × 10 cm to 9 cm × 9 cm × 4 cm) half full of river sand (**ø < **0.2 mm) were put on the bottom of the tanks. Many small boxes (0.7–1.3 boxes for each male in the tank) worked better in tanks with more than four males. The boxes were put in the fish tanks for 2–3 h 3 to 5 days a week, and the average number of eggs laid by each female in the sand was between 20 and 50. The sand boxes were always added to the tanks at the same hour of the day (for example, from 10 am to 1 pm, or from 2 to 5 p.m.), to train the fish to breed at those hours and to maximize egg production.

### Egg collection

Eggs were collected by removing the boxes from the tank and pouring the sand through a strainer (1-mm grid width). Eggs retained in the strained were then transferred to a petri dish filled with aquarium water.

Once collected, dead and mis-shaped eggs were removed and the fertilized eggs were transferred to another petri dish in 35 mL of aquarium water at a maximum density of 50 eggs per petri dish.

The eggs were then kept at 26 °C.

### Egg husbandry

Embryos, wild-type fish and F1, F2 or F3 transgenic embryos were kept in 50-mL petri dishes in 35 mL of tap water, at 26 °C, until their eyes turned from colourless to black (that usually required around 14 days). During this period, the water in the petri dish was replaced once a day with new water and the embryos that died during development were removed daily.

Once the eye of the surviving embryos turned black, they were moved to peat moss petri dishes (50 mL petri dishes with 0.5 cm of fairly humid peat moss or coconut fibre pressed on the bottom). Up to 150 embryos were put in each peat moss petri dish and kept at 26 °C in this condition until their eyes turned from black to totally golden (that usually required 14 days).

Once golden-eyed, the embryos were transferred in small 1-L boxes filled for 2/3 with 4 °C aquarium water and 1/3 with a 4 °C humic acid solution (humic acid from sigma Cat. No. 53680-10G, 1 g dissolved in 1 L of aquarium water). In these conditions, usually more than 75% of the embryos were able to hatch in 24 h.

To force the hatch of the remaining not-hatched embryos, we availed of a not yet published technique that anyway in our hands worked 100% of the time.

The eggs that were not able to hatch in 24 h were collected in a 50-mL falcon tube filled with aquarium water and kept at 18–23 °C on the bench with the falcon tube standing and without the lid. With this procedure, usually between 80 and 100% of the eggs were able to hatch within 18 h.

The hatched fish were then transferred in the 1-L box (the same where they hatched), and the boxes were put in an incubator with a temperature between 26 and 28 °C, a light cycle of 12-h light and 12-h darkness and an air tube providing a constant and moderate air influx in the boxes.

Everyday half of the solution in each hatching box was removed and replaced with autoclaved aquarium water and the bottom of the boxes cleaned from any debree, shell fragment or food leftover.

After the first week, larvae were moved to small 0.8-L tanks in the general system, at a density of 5 to 7 larvae per tank. Fish were kept in these conditions for 1 week and at the age of 2 weeks after hatch were split singularly in 3-L tanks.

### Transgenic eggs husbandry

*Nothobranchius furzeri* F0 transgenic embryos (freshly injected embryos) were kept in a 50-mL petri dish in 35 mL of system water, at 26 °C for the first 2 days after injection. During this period, the water in the petri dish was replaced once a day and the embryos that died were removed daily.

After 2 days, surviving embryos were moved to peat moss petri dishes (50 mL petri dishes with 0.5 cm of fairly humid peat moss or coconut fibre pressed on the bottom). Up to 150 embryos were put in each peat moss petri dish and kept at 26 °C in this condition until their eyes fully developed turning to a totally golden colour. (This required from 20 days to several months since a lot of injected embryos entered diapause II.) Once golden-eyed, the embryos were treated like the others described previously.

After the fish reached the second week of life, just before splitting them in singular tanks, they were screened with a fluorescence microscope to check the reliability of the signal, and the embryos with an absent, too weak or not correct signal were discarded.

### Transgenic embryo screening

Transgenic embryos were screened with a fluorescence microscope at different stages (epiboly, dispersed phase, mid-somitogenesis, hatched larvae or 4-week-old fish) depending on the injected construct.

Fish were anesthetized with Tricaine 0.5X for few minutes, then screened and selected.

Fish that showed the correct pattern of expression of the transgene were raised; the remaining fish were discarded.

All the embryos belonging to all the transgenic generations were screened.

### Transgenic lines generation

#### FUCCI plasmids construction

FUCCI plasmids were constructed starting from zebrafish mKO2-zCdt1(1/190) and mAG-zGem(1/100) plasmids, replacing the original promoter with the zebrafish ubiquitin promoter.

Original plasmids were amplified in *E. coli* and purified with Wizard^®^ Plus SV Minipreps DNA Purification kit from Promega. Then, 2 μg of each plasmid was cut with Nhe1 and BamHI, ran on an agarose gel, and the higher molecular mass band was purified using Wizard^®^ SV Gel and PCR Clean-Up kit from Promega.

Ubiquitin promoter was amplified by PCR from pENTR5′_ubi using Q5^®^ High-Fidelity DNA Polymerase, with these primers (F: 5′-cattgaGCTAGCatggatgttttcccagtcacgacg-3′, R:5′-tgactaGGATCCtgtaaacaaattcaaagtaagat-3′) and the following thermocycling protocol: 98 °C 30′′ (98 °C 10′′, 52 °C 30′′, 72 °C 2′) X35 cycles 72 °C 2′ pENTR5′_ubi was a gift from Leonard Zon (Addgene plasmid # 27320).

The PCR product was ran on an agarose gel and the band-purified using Wizard^®^ SV Gel and PCR Clean-Up kit from Promega.

Vectors and the ubiquitin promoter insert were ligated over night using NEB T4 DNA Ligase, in a molar ratio of 1:3, mixing 50 ng of vectors and 73 ng of insert.

The resulting plasmid was then amplified in *E. coli* and purified with Wizard^®^ Plus SV Minipreps DNA Purification Promega. This final plasmid was injected into 1-cell stage embryos together with the tol2 synthetic RNA.

#### Tol2 RNA synthesis

Tol2 synthetic RNA was synthesized using the mMESSAGE mMACHINE^®^ SP6 Transcription Kit from Ambion. pCS2FA-transposase plasmid, linearized using NotI, was used as template and sp6 as promoter for RNA transcription. Resulting synthetic RNA concentration was measured with a nanodrop and on an agarose gel.

### Eggs injection

Transgenic fish were generated injecting 1 μL of a solution containing 30 ng/μL of TOL2 RNA, 40 ng/μL of plasmid DNA, 400 mM of KCl in 1 cell stage *N. furzeri* embryos.

Injections were performed at 26 °C using a Leica M80 Stereo Microscope and a Tritech air injection system.

Eggs were oriented on a 2% agar framework and injected sequentially.

Once injected, eggs were put in a petri dish with 26 °C aquarium water. After 1.5 h, the dead eggs were removed, and the others were allowed to develop in a new petri dish with 35 mL of 26 °C aquarium water.

### Microscopy

#### Samples preparation

All the pre-hatching embryos acquired were prepared in this way, regardless of their developmental stage:

#### Bright-field acquisitions

Several eggs (averagely 4) were put in a 1.5-mL falcon tube with 10 mL of liquid low melting agarose 1.5% solution, not warmer than 32 °C. The falcon was left in agitation for 30 s to completely mix the eggs and the liquid agarose.

The eggs and 3 mL of the agar solution were poured in a Willco dish, and the eggs were put in the middle using forceps, spaced each other by more or less 2 mm. After pouring eggs into the dish, the eggs were carefully oriented in the desired way using forceps, and then, the agar was left solidify at room temperature for 10 to 30 min.

Once ready with agarose embedded eggs, the Willco dish was parafilmed, reversed upside down and put under a Leica M80 stereo microscope. Up to 6 eggs were embedded each time for bright-field acquisitions. The microscope was set up to offer the best condition of brightness and contrast, and the zoom was adjusted accordingly to the number and the size of the eggs to image.

Photographs were captured every 5 min with a Nikon Digital Sight DS-Fi1 camera or with a ZEISS Axiocam ERc 5-s camera. Acquisition lasted several days.

#### Bright-field videos and images processing

All the acquired photographs relative to a single acquisition session were loaded on Fiji ImageJ as an image sequence and analysed to verify synchrony between the eggs in the acquisition field.

A single egg present in the image was chosen, rotated and cropped, in order to make a new image sequence including only that embryo, and this was saved in a separate folder.

This new folder was imported in Sony Vegas, a video-make software, in order to make a smooth video from a discontinuous image sequence. Time, writings, video effects and soundtrack were added as different levels, and all the levels were rendered only once in a.avi file to maintain the best possible resolution.

Bright-field images were edited with GIMP. Contrast, brightness and sharpness were modified in order to make pictures the most possible beautiful, clear and informative.

#### Confocal acquisitions

For confocal acquisitions, a Leica TCS SP5 X inverted microscope was used. Three to five embryos were embedded in agar in a Willco dish that was then sealed with parafilm. The position of each embryo in the dish was marked with the Leica confocal software Leica LAS X Core, and automatically, every 10 min, every embryo in the dish was scanned sequentially with a 488-nm and a 543-nm argon laser. Only the top half of each embryo (about 500 μm) was scanned, since the light penetration is limited in the lower half, resulting in distorted and faded lower part images. Image stacks were acquired every 7–12 μm, depending on samples and on experiments. Experimental sessions lasted from 10 to 61 h, depending on the developmental stage acquired. Time lapse acquisitions were performed at 26 °C. Images were acquired with a 10× dry objective and with a digital zoom of 1.2×.

#### Fluorescence image processing

All the stacks relative to a single time point were projected using Fiji Z-project standard deviation algorithm, both for green and for red fluorescence channels. Projected images were then adjusted in brightness and contrast with GIMP in order to optimize the “signal-to-noise” ratio and be more clear and informative.

#### Imaris analysis

Raw data from time lapse acquisitions obtained with the Leica confocal microscope were analysed with Imaris. Whole time lapse acquisition data sets were loaded on Imaris; then, the red and green channels were adjusted in brightness and contrast in order to separate the cell nuclei from the background as best as possible. For the stages of epiboly, diapause I and the dispersed phase, the red and green nuclei were then converted in EVL cell dots, epiblast red cells and epiblast green cells with Imaris particle analysis function. It was possible to separate EVL red cells and epiblast red cells (that appeared both red) using the size recognition function of Imaris particle analysis.

For the stages of reaggregation, axis formation, segmentation and diapause II, it was not possible to track separately the cell nuclei, so the aggregates of cells were converted in a unique surface with the surface analysis function of Imaris. Parameters for particle or surface recognition were adjusted in different ways for each set of images analysed, in order to track the structures of interest getting rid of the background and non-specific signals.

Particles belonging to background or to artefact structures that were not filtered out by the automatic recognition process were removed manually.

#### Single cell suspension preparation and FACS analysis

Two-to-four 1-week-old embryos (euthanized with Tricaine) or two gonads extracted from 8-week-old adult fish were carefully dried with paper towel and washed twice for 2 min with PBS 1X in an Eppendorf tube. PBS was replaced with a mix of 460 μL 0.25% trypsin–EDTA+ 40 μL collagenase 100 mg/mL. Samples were kept in this solution at a temperature of 30 °C and harshly pipetted for 10 to 30 min, until complete dissociation. Once dissociated in a single cell suspension, 800 μL of DMEM-10% FBS was added to the tube, and the solution was mixed and centrifuged at 500*g* at RT for 5 min. Supernatant was discarded, and the cells were washed once with 750 μL of PBS 1X. The tubes were centrifuged at 500*g* at RT for 5 min and the supernatant discarded and replaced by 300 μL of PBS 1X+ 6 μL of Hoechst 33342. The samples were resuspended, incubated 15 to 30 min at RT and then analysed.

FACS analyses were performed using a BD LSRFortessa analyser and the software BD FACSDiva™. The fluorochromes or dyes detected were Kusabira-Orange, Azami-Green, Hoechst and propidium iodide.

Final FACS results were analysed using FlowJo.

#### Graph production

Imaris-related graphs, concerning FUCCI fluorescence analysis, were made with Imaris, with the particle or surface analysis feature. All data plotted in the graphs are relative to the cleavage analysis or the track analysis done by the program.

## Supplementary information

**Additional file 1: Movie S1.** Movie of particle tracking during epiboly.

**Additional file 2: Movie S2.** Bright-field time lapse of early dispersed phase.

**Additional file 3: Figure S1.** Graph depicting the changes of fluorescence in four embryos released from diapause I.

**Additional file 4: Movie S3.** Time lapse fluorescent imaging of reaggregation phase, axis formation and early somitogenesis.

**Additional file 5: Figure S2.** Still fluorescent image of a direct-developing embryo.

**Additional file 6: Movie S4.** Time lapse fluorescent imaging of exit from DII.

**Additional file 7: Movie S5.** Time lapse, comparison of four different embryos that exit from DII.

**Additional file 8: Figure S3.** Quantification of the fluorescence of the four different embryos shown in Additional file 7.

## Data Availability

All data generated or analysed during this study are included in the additional information files or can be obtained by the corresponding author on a reasonable request.

## Abbreviations

EL: Epiblast layer. A layer of cells composed by blastomeres that divides actively during development and will take part in the generation of the several embryonic and fish major structures like head tail trunk and organs
EVL: Enveloping layer. A thin layer of cells that envelopes all the embryo. It is the most external layer. The cells belonging to this layer are big with big nuclei that do not divide
FUCCI: Fluorescent Ubiquitination-based Cell Cycle Indicator
DI: Diapause I. A dormancy stage peculiar of annual killifish species that occurs after the completion of epiboly, during the dispersed phase
DII: Diapause 2. The second and most important dormancy stage of annual killifish species. Fish can stop in DII only entering a different developmental trajectory after the reaggregation phase. The final developmental block occurs at the mid-somitogenesis stage
DC: Diapause Committed embryo. An embryo that undertook the diapause II trajectory of development and that will stop for sure in diapause II during the somitogenesis stage
DD: Direct-Developing embryo. An embryo that is following the not diapause II developmental trajectory. These embryos grow more in lateral size during somitogenesis and never stop their development in this phase
WS: Wourms’ Stage. Developmental stage referring to the embryonic description made by Wourms for the killifish species *Austrofundulus limnaeus*
YSL: Yolk syncytial layer. A layer of cells that form a syncytium and that are in direct contact with the yolk. This is the most internal layer, through this layer nutrients from the yolk can be delivered to the upper layers
