# Supplementary figures and images for: Cell cycle dynamics during diapause entry and exit in an annual killifish revealed by FUCCI technology
Source: EvoDevo. 2019 Nov 8;10:29. doi: 10.1186/s13227-019-0142-5 (PMC6842169; doi:10.1186/s13227-019-0142-5)

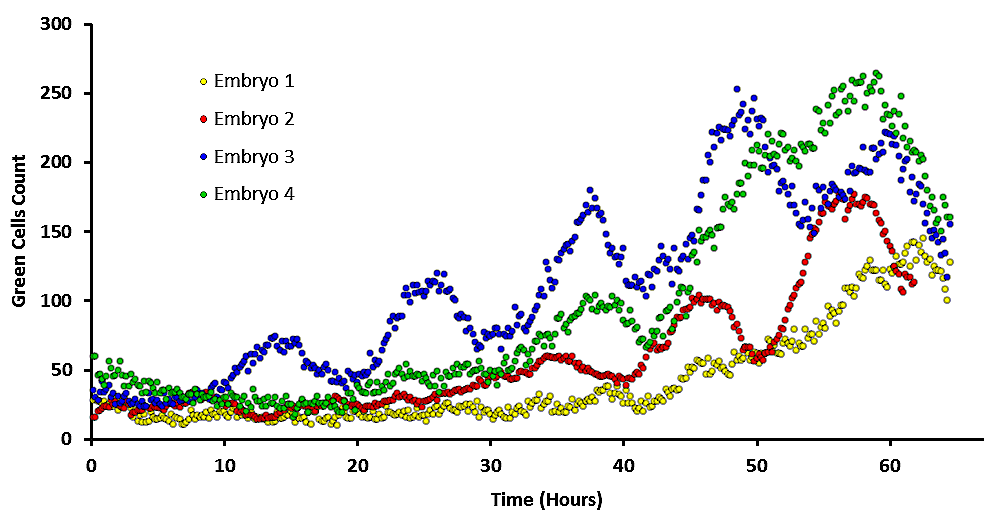

Supplement: Supplementary file 3 — Additional file 3: Figure S1. Graph depicting the changes of fluorescence in four embryos released from diapause I. [file 13227_2019_142_MOESM3_ESM.tiff]

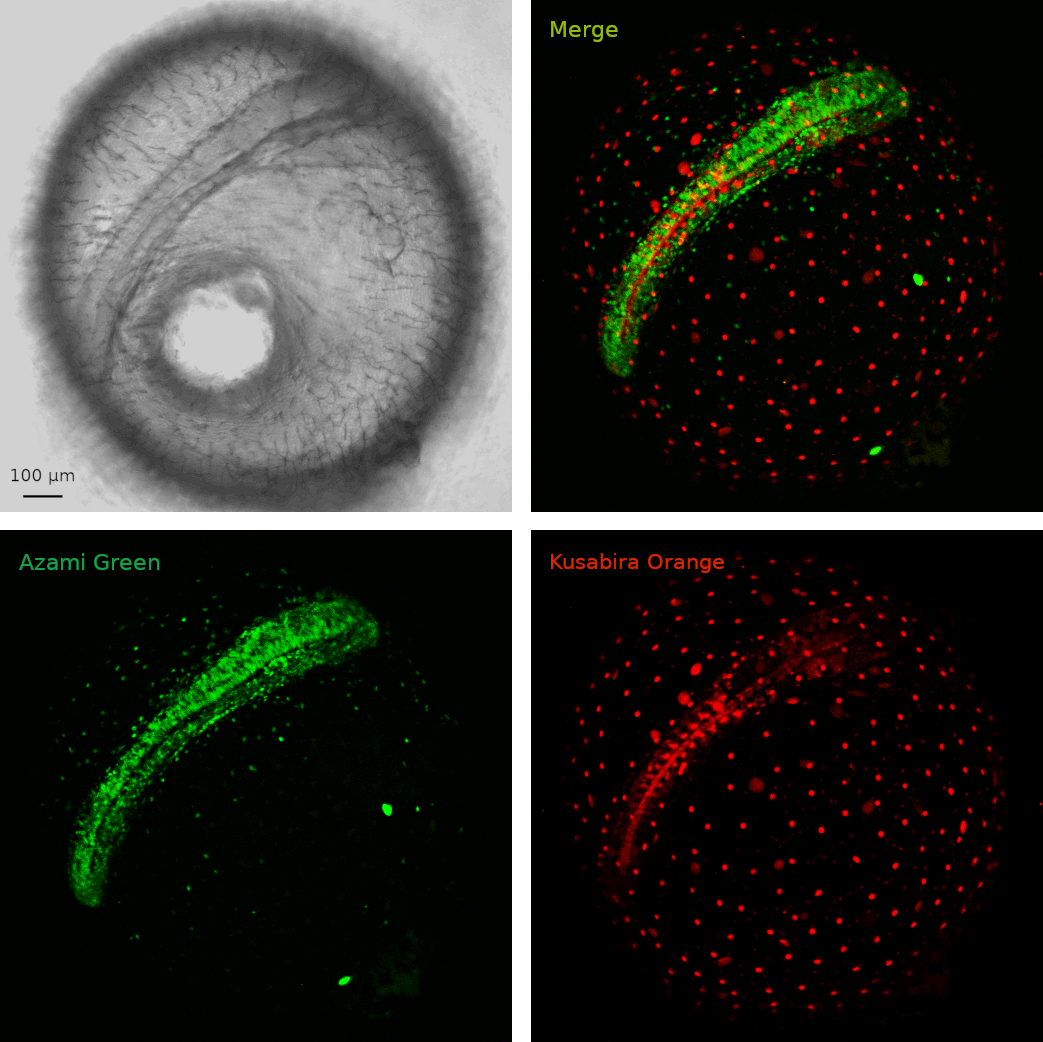

Supplement: Supplementary file 5 — Additional file 5: Figure S2. Still fluorescent image of a direct-developing embryo. [file 13227_2019_142_MOESM5_ESM.tiff]

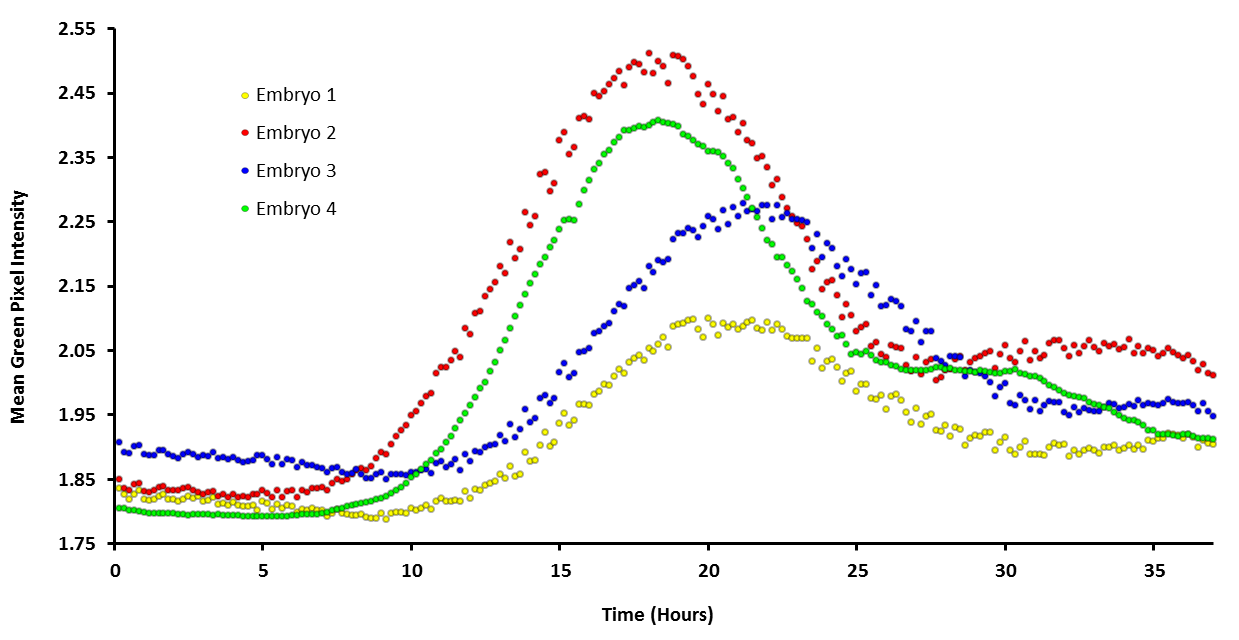

Supplement: Supplementary file 8 — Additional file 8: Figure S3. Quantification of the fluorescence of the four different embryos shown in Additional file 7. [file 13227_2019_142_MOESM8_ESM.tiff]
